# Suppression of *NRAS*-mutant melanoma growth with NRAS-targeting Antisense Oligonucleotide treatment reveals therapeutically relevant kinase co-dependencies

**DOI:** 10.1038/s43856-025-00932-5

**Published:** 2025-06-05

**Authors:** Valentin Feichtenschlager, Yixuan James Zheng, Tiange Qu, Dasha Hohlova, Ciara Callanan, Linan Chen, Christopher Chen, Wilson Ho, Albert Lee, Yeonjoo Hwang, Arowyn Courtright, Thy Nguyen, Olivia Marsicovetere, Denise P. Muñoz, Klemens Rappersberger, Jean-Philippe Coppe, Susana Ortiz-Urda

**Affiliations:** 1 Department of Dermatology, Mt Zion Cancer Research Center, University of California San Francisco, San Francisco, CA USA; 2 Department of Dermatology, Clinic Landstrasse Vienna, Academic Teaching Hospital, Medical University Vienna, Vienna, Austria; 3 School of Medicine, University of California San Francisco, San Francisco, CA USA; 4 Department of Orofacial Science, Health Science West, University of California San Francisco School of Dentistry, San Francisco, CA USA; 5 Department of Biology, University of San Francisco, San Francisco, CA USA; 6 Department of Hematology-Oncology, Helen Diller Family Comprehensive Cancer Center, University of California San Francisco, San Francisco, CA USA; 7 Department of Radiation Oncology, Helen Diller Family Comprehensive Cancer Center, University of California San Francisco, San Francisco, CA USA

**Keywords:** Melanoma, Targeted therapies, Cell signalling, Melanoma

## Abstract

**Background:**

Melanoma is an aggressive form of skin cancer, and patients with *NRAS*-mutant melanoma face limited treatment options due to the lack of direct NRAS inhibitors. This study explores the utilization of antisense oligonucleotides (ASOs) to directly target *NRAS*-mRNA for therapeutic approaches.

**Methods:**

We designed and tested *NRAS*-mRNA-targeting ASOs. Experiments in melanoma cell lines and mouse models assessed effects on cell survival, apoptosis, and tumor growth. A kinase activity profiling platform identified therapeutically exploitable pathways influenced by NRAS suppression.

**Results:**

Our research suggests that ASOs do not need to target the mutated NRAS segment to be effective. ASOs designed for the non-mutated NRAS sequence eliminate NRAS-dependent melanoma cells while sparing NRAS wild-type cells. They act independently of subcellular target localization, reduce NRAS-mRNA levels, inhibit MAPK signaling, induce apoptosis, and suppress melanoma growth in vitro and in vivo. Outcomes of high-throughput kinase activity mapping (HT-KAM) indicate a significant dependency between *NRAS*-mRNA expression and the activity of MEK1, FGFR2, and CDK4 kinases. Co-targeting these kinases enhances the antiproliferative effect of NRAS ASOs, showing synergy.

**Conclusions:**

These findings highlight antisense oligonucleotides as a promising therapeutic approach for *NRAS*-mutant melanoma. By effectively blocking *NRAS*-mRNA, this strategy overcomes challenges posed by the absence of a direct small molecule inhibitor for NRAS, and may offer new treatment options for patients.

## Plain Language Summary

Melanoma is the most dangerous type of skin cancer. Some people develop melanoma due to a change (mutation) in their DNA in a gene named *NRAS*. Changes in *NRAS* lead to cancer growth by causing a protein called NRAS to be continuously active. These patients have limited effective treatment options. Therefore, we explored a treatment that can prevent cancer cells from producing the NRAS protein. This treatment effectively killed melanoma cells whilst sparing healthy cells and reduced melanoma growth in a mouse model. It also enhanced the effect of other cancer medications. Our treatment strategy demonstrates that these people with melanoma could potentially benefit from similar therapies in the future.

## Abstract (web summary)

Feichtenschlager et al. explore antisense oligonucleotides (ASOs) targeting *NRAS*-mRNA as a therapeutic approach for *NRAS*-mutant melanoma. Their findings demonstrate that the ASOs effectively eliminate *NRAS*-dependent melanoma cells, inhibit MAPK signaling, and synergize with kinase inhibitors, offering a promising strategy for clinical applications.

## Introduction

Melanoma is the deadliest form of skin cancer, with continuously rising incidence rates in the past decades^1^. Activating mutations in *RAS* oncogenes of small GTPases are one of the most common cancer driving mutations, being detected in a third of all human cancers^2^. In melanoma, mutations in the *RAS*-isoform neuroblastoma *RAS* viral oncogene homolog (*NRAS*) are found in approximately 25% of cases, being the second most frequent mutation type after v-raf murine sarcoma viral oncogene homolog B1 (*BRAF*)^3^. Oncogenic *NRAS* missense mutations at codons 12, 13, or 61 induce constitutive NRAS activity, with mutations in codon 61 occurring in 90% of *NRAS*-mutant melanoma^3^. NRAS-driven melanomas are characterized by unique clinical features, such as thicker tumors, higher rates of occurrence on extremities, rapid onset of treatment resistance, and higher mitotic indices. Ultimately these factors lead to poor prognosis^3–6^. Efforts to develop effective drugs targeting RAS proteins have faced substantial challenges^7^, leading to programs such as the “RAS Initiative”; a key initiative by the National Cancer Institute (NCI) to explore novel therapies for RAS-related cancers (source: https://www.cancer.gov/research/key-initiatives/ras). The challenges of RAS-targeting are primarily attributed to the exceptionally high affinity of RAS proteins for GTP binding and the absence of accessible binding sites^8^. Currently, immunotherapy with anti-PD-1 checkpoint inhibitors stands as the primary pharmacological treatment for *NRAS*-mutant melanoma, albeit offering patients only modest benefits^9,10^. Alternative strategies encompass monotherapy and drug combinations targeting the mitogen-activated protein kinase signaling pathway (MAPK, also recognized as RAS-RAF-MEK-ERK), as well as the parallel PI3K/AKT pathway. Both RAS-mediated downstream effector pathways share closely interconnected regulatory mechanisms, ultimately contributing to the cellular survival of *NRAS*-mutant melanoma cells^3^. Patients with *BRAF*-mutant melanoma benefit from targeted and immunotherapy to a higher extent compared to patients with *NRAS*-mutant melanoma^3^, which underscores the necessity for novel targeted therapies and innovative drug combinations.

An emerging field in drug development is centered on targeting RNAs with antisense drugs, particularly Antisense Oligonucleotides (ASOs) and small interfering RNAs (siRNAs)^11^. Despite their shared function to bind RNA through Watson–Crick base pairing, ASOs and siRNAs exhibit distinct mechanisms to modulate gene expression^12^. SiRNAs are double stranded, with one strand getting lost (passenger), and the other strand (guide) initiating RNA-degradation through interacting with the RNA-induced silencing complex (RISC)^12,13^ ASOs are single-stranded DNA oligonucleotides that can be used to effectively silence gene expression in both the nucleus and the cytoplasm through RNase H1-mediated RNA depletion^12,14–16^. Furthermore, the incorporation of additional chemical modifications such as “GapmeR” structures can enhance nuclease protection and target binding specificity of ASOs. A prevalent GapmeR modification in ASOs involves the use of locked nucleic acids (LNA®) at the flanking ends of the RNA-targeting DNA sequence, thereby improving target affinity and stability^17,18^. ASOs are undergoing testing in pre-clinical models and clinical trials, with an increasing number gaining approval from regulatory bodies such as the FDA and EMA for a range of multi-drug and mono-therapeutic applications^19^. Notably, ASO mediated targeting of the RAS gene family member KRAS has displayed promising results in pre-clinical studies aimed at KRAS-dependent tumors^20^.

Here, we present on the efficacy of targeting *NRAS*-mRNA using GapmeR ASOs as a highly selective and efficient approach to combat *NRAS*-mutant melanoma. Previous efforts to target *NRAS*-mRNA in *NRAS*-mutant melanoma focused on the mutational site of the *NRAS*-mRNA sequence^21,22^. Our approach demonstrates that NRAS-mutant melanoma cells can be specifically targeted by exploiting the full sequence, allowing to target various NRAS-mutant melanoma subtypes with the same ASO sequence. Addressing this critical vulnerability offers more flexibility in ASO development for designing NRAS-targeting therapies not restricted to the mutational site. NRAS ASO treatment induced apoptosis and robustly decreased cell growth and colony formation in *NRAS*-mutant melanoma cell lines in vitro, without significantly affecting the growth of non-malignant *NRAS* wild type (WT) cell lines. In an in vivo setting, NRAS ASO treatment effectively reduced tumor growth in mice harboring *NRAS*-mutant melanoma xenografts, all while displaying no apparent toxic side effects. Using a high-throughput kinase activity profiling platform allowed us to reveal specific kinase signaling vulnerabilities that emerge upon NRAS ASO treatment, offering opportunities for combination therapy with kinase inhibitors to achieve synergistic antiproliferative effects. Our findings establish that NRAS can be broadly, directly, and efficiently targeted with ASOs in *NRAS*-mutant melanoma, potentially opening new therapeutic avenues for melanoma patients.

## Methods

### *NRAS* dependency analysis

The cell line dependency data were downloaded from the Broad Institute Dependency Map portal (DepMap) website (https://depmap.org/portal/download/custom/). Dependency of cell lines to *NRAS* transcription was tested using the DepMap CRISPR (DepMap 22Q1 Public+Score, Chronos, *n* = 1150) and RNAi (Achilles+DRIVE+Marcotte, DEMETER2, *n* = 710) dependency scores (probability of dependency). Gene dependency was measured by a decrease in, or stasis of cell viability after gene perturbation with CRISPR or RNAi, collectively termed “gene effect”^23^. For each gene examined, the probability of dependency is calculated using a project Achilles Beyesian inference method that determines the probability that the gene of interest can be considered an essential gene rather than a nonessential or non-expressed gene^23^. A dependent cell line is defined as one that has a probability of dependency greater than 0.5^23^. Finally, individual gene scores were plotted along a distribution showing the *NRAS* gene effect across all cell lines examined.

### Cell culture

Cell lines VMM39, H929 and SW1271 were acquired from American Type Culture Collection (ATCC®). Human melanoma cell-lines D04, MM415, WM3629, Sk-Mel-2, WM3060, and WM1366 were a generous gift by Dr. Boris Bastian at the UCSF. The human melanoma cell-line NZM40 was gifted by Dr. Rony Francois at the UCSF. Primary human melanoma (Hs852T) and liver (Hs775Li) cell lines were acquired from the Cell and Genome Engineering Core (CGEC) at UCSF. Primary human melanocytic cell-lines (PHM), derived from infant foreskin of healthy donors were sourced from the Ortiz’ lab cell repository. Melanocytes were cultured in M254 medium supplemented with Human Melanocyte Growth Supplement (HMGS, 1x final solution), Hs775li cells were maintained in DMEM H21 medium containing 10% (v/v) heat-inactivated fetal bovine serum (FBS), while FHC cells were grown in DMEM F12 medium with 10% (v/v) heat-inactivated FBS. All other cell lines were maintained in RPMI 1640 medium supplemented with 10% (v/v) heat-inactivated FBS. All cultures were incubated at 37 °C in a humidfied atmosphere with 5% CO2. Cell culture related research recieved approval from the UCSF Human Research Protection Program Institutional Review Board (IRB# 12-0948) and was performed in accordance with relevant guidelines and regulations. The drug resistant cell lines (RM suffix) were established as previously described^24^, and chronically exposed to trametinib (D04RM: 5 nM, MM415RM: 55 nM, WM3629RM: 14 nM, and Sk-Mel-2RM: 7 nM). Cells were tested for mycoplasma and cell line authentication was performed by vendors.

### Purification of nuclear and Cytoplasmic RNA

Total nuclear and cytoplasmic material wwas extracted using the SurePrep™ Nuclear/Cytoplasmic RNA purification kit (Norgen Biotek Corp®, cat.no.: 21000) following the manufacturer’s instructions.

### In situ hybridization and immunofluorescence

In situ hybridization analyses were performed using the RNAscope Multiplex Fluorescent Reagent Kit version 2 system, following standard protocol, and customized probes to *NRAS-*mRNA. After RNAscope, sections immediately underwent immunofluorescent staining with NRAS-binding antibodies. Immunofluorescent staining was executed using the following conditions: Primary antibody dilutions of 1:150 or 1:50 for NRAS ProteinTech 10724-1-AP, or LsBio LS-C174539, respectively. Overnight incubation was performed at 4 °C. Secondary antibody treatment of the sections was performed using goat anti-mouse AlexaFluor-Plus 555 (1:400, Invitrogen) or donkey anti-rabbit AlexaFluor-Plus 488 (1:400; Invitrogen) and DAPI (2 h prior to imaging). To amplify the fluorescent signals for NRAS, TSA Opal520 fluorophores were used, in accordance with the instructions in the Multiplex Fluorescent Reagent Kit version 2. Cells were treated with 100 nM final ASO concentration for 1 day.

### Fluorescence imaging

A Zeiss Axio Observer Z1 was used for fluorescence imaging (20X objective). Throughout the process, images were captured at a constant exposure, using identical microscope settings.

### Fluorescence quantification

Subcellular quantification defined by DAPI staining and fluorescent signal of NRAS-binding RNAscope probes was performed using QuPath-0.3.2. Punctua per cell were calculated following a protocol for quantitative Analysis of Gene Expression in RNAscope processed samples by Secci et al.^25^.

### RNA secondary structure

RNA secondary structures and minimum free energy (MFE) structures were analyzed using the RNAfold Web Server (University of Vienna, http://rna.tbi.univie.ac.at/cgi-bin/RNAWebSuite/RNAfold.cgi)^26^. A dynamic programming algorithm described by Zuker et al.^27^ was used to predict the MFE.

### On- and off-target binding affinity analysis of NRAS ASOs

BLAST by the National Library of Health (https://blast.ncbi.nlm.nih.gov) was used to screen for ASO targets in the human, respectively mouse transcriptome and binding affinity. The human and mouse genomic + transcript database was screened with program selection “somewhat similar sequences (blastn). Targets on the Plus/Minus strand were considered as potential targets and models (XM/XP) were excluded from the analysis.

### Oligonucleotide transfection

Affinity Plus^TM^ (LNA) antisense oligonucleotides were purchased from IDT and used for all described in vivo and in vitro experiments. For non-targeting control ASO design, the sequence 5’-AACACGTCTATACGC-3’ was used. ON-TARGETplus SMARTpool siRNA (NRAS siRNA) was purchased from Dharmacon^TM^. Cells were seeded 24 h prior to transfection and the transfection reagent Lipofectamine^TM^ 3000 (2ul/ml) was added according to the manufacturer’s instructions.

### RNA extraction and quantitative real-time PCR (qRT-PCR)

Total RNA was extracted from cells and tissues using TRIzol™ Solution (Thermo Fisher Scientific®), Phenol:chloroform:isoamyl alcohol (MilliporeSigma®) or NucleoSpin® RNA kit (Takara Bio USA, Inc, following the manufacturer’s instructions. NanoDrop™ ND-1000 (Thermo Fisher Scientific®) or Quibit™ 4 (Thermo Fisher Scientific®) was used for quantification of total RNA. Reverse transcription of 50 ng of RNA was performed using the cDNA synthesis and gDNA removal QuantiTect® Reverse Transcription Kit (Thermo Fisher Scientific®). The iTaq^TM^ Universal SYBR® Green Supermix (Bio-Rad Laboratories, Inc.), 20 ng of cDNA, and the QuantStudio^TM^ 5 Real-Time PCR System (Thermo Fisher Scientific®) was used for Real time PCR analysis. Calculations for relative gene expression were performed following the comparative Ct method, normalized to *Β-ACTIN*. Primers were obtained from IDT and are listed in Supplementary Table 9.

### Protein extraction and immunoblotting

One day prior to transfection, cells were seeded in six well-plates. Homogenization of total protein lysates was performed in 1x RIPA buffer and Halt protease and phosphatase inhibitor cocktail (Thermo Fisher Scientific®) followed by centrifugation at 14,000 RPM/minute at 4 °C. Pierce™ BCA Assay Kit (ThermoFisher Scientific®) was used to quantify protein concentrations, followed by linear absorbance measurement using the Synergy™ HT (Agilent Technologies Inc) plate reader. Total protein dissolved in 1× Laemmli buffer (10% 2- mercaptoethanol) was separated by SDS/PAGE, followed by transfer to a PVDF membrane (IPVH00010; MilliporeSigma®) through electroblotting with 20% (v/v) methanol. Blocking was performed for 1 h in in Intercept (TBS) blocking buffer (LI-COR®). Overnight incubation of membranes was performed at 4 °C with primary antiserum for NRAS (Santa Cruz Biotechnology®, cat.no.: sc-31, dilution 1:50), ERK1/2 (Cell Signaling Technology®, cat.no.:4695, dilution 1:600), p-ERK1/2 (Cell Signaling Technology®, cat.no.:4370, dilution 1:600), GAPDH (Cell Signaling Technology®, cat.no.:97166, dilution 1:1,000), p-S6 (Cell Signaling Technology®, cat.no.:4857, dilution 1:500), S6 (Cell Signaling Technology®, cat.no.:2217, dilution 1:600), B-ACTIN (Cell Signaling Technology®, cat.no.: 8457, dilution 1:3,000, or abcam, cat.no.:8226, dilution 1:1,000), p-Akt (Cell Signaling Technology®, cat.no.:4060, dilution 1:400), and Akt (Cell Signaling Technology®, cat.no.: 9272, dilution 1:400) following incubation with secondary Goat Anti-Rabbit and Anti-Mouse serum (LI-COR®, dilution 1:5,000) for 1 h. Membranes were scanned using the Li-COR® Odyssey® Imaging system. Quantification of protein expression was performed using Image Studio Lite Version 5.2.5.

### Cell growth analysis

Cells were seeded in 96 well-plates one day prior to treatment (seeding density was dependent on cell doubling time, ranging 0.7-2 × 10^^3^ cells/well). Cells then were treated with ASOs for five days unless specified otherwise. Cell growth analysis was performed with Promega® CellTiter-Glo® and total luminescence was measured on a plate reader (Synergy™ HT, Agilent Technologies Inc, Gen5 software). Control ASO treatment was used for normalization.

### Colony formation

Experiments were performed in duplicates in 6 cm well plates. Cells were seeded at low density one day prior to transfection with a final concentration of 50 nM of either NRAS ASO or Control ASO. Seven days after treatment, cells were fixed, stained with 0.1% crystal violet. Colony counts were gathered by using a reference colony (~50 cells), only counting colonies that were the same size or larger. Colony counting was performed by three individual people.

### Annexin V assay

One day prior to transfection D04 cells (seeding density: 1 × 10^^5^) were seeded in six well-plates. After one day of ASO incubation live, dead, and apoptotic cells were differentiated (Invitrogen™ Dead Cell Apoptosis Kits with Annexin V, cat.no.: V13241), following the manufacturers instructions. Sorting of cells was performed using a BD® LSR II Flow Cytometer.

### Caspase Glo 3 & 7 assay

Cells were seeded in 96 well-plates one day prior to transfection and seeding density was dependent on cell doubling time (2–3 × 10^^3^). After one day ASO incubation total luminescence was measured on the Synergy™ HT (Agilent Technologies Inc) plate reader (Promega® Caspase-Glo® 3/7 Assay and Gen5 software).

### Animal models

The Office of Research institutional Animal Care and Use Program (IACUC, Chair: Jeremy Lieberman, MD) at the University of San Francisco (UCSF) approved the rodent experimental procedures. All in vivo studies were performed under an authorized protocol number (AN174613-03). Maintenance of mice was performed in a pathogen free environment and all animals consistently had access to food and water. Mice were obtained from JAX® and subcutaneous injections, located at the posterior dorsal flanks of 4- to 6-week-old homozygous nude Foxn1^nu^/Foxn1^nu^ mice (Stock.no 007850) were performed using 2 × 10^^6^ D04 cells in 150 µl of PBS and 50 µl of Matrigel. Mice were randomly assigned to treatment groups to minimize bias and investigators were blinded for tumor volume assessment. Group sizes were based on prior in vivo experiments demonstrating reproducible treatment effects under similar conditions. Only female mice were used to reduce sex-related confounders and ensure consistency across experimental groups. A digital caliper and the formula 0.5 x (length x (width^2)) was used to measure and calculate tumor volume. Treatments were applied 3x/week with 200 µg of ASOs diluted in an overall amount of 100 µl PBS. ASOs were injected subcutaneously into the dorsal region. Mice were continuously observed for signs of distress (e.g., increased aggression or withdrawal, weight loss, changes in grooming habits, vomiting, diarrhea, abnormal respiratory patterns) or disorder (e.g., abnormal posture, loss of coordination, tremors). Mice were euthanized at the desired endpoint of the experiment. After euthanasia, tumor samples were excised, immediately placed in RNAlater™ Stabilization Solution (Thermo Fisher Scientific®) and stored at −20 °C. Blood was collected from mice via cardiac puncture and serum was analyzed by the clinical laboratory at Zuckerberg San Francisco General Hospital for liver panel parameters (project ID: 7001137). All experiments were performed in accordance with the Laboratory Animal Resource Center (LARC) guidelines at UCSF.

### Kinase activity mapping technology

Treamtment of cells was performed with ASOs for 24 h. At ~85% confluency cells were washed three times with cold PBS and lysed with freshly prepared 1X cell lysis buffer (1 ml per 2.5 × 10^6^ cells, 10x Cell lysis buffer, Cell Signaling Technology®, cat.no.: 9803), complemented with 1x Halt Protease and Phosphatase (Thermo Fisher Scientific® cat.no.: 1861281). The lysates were scraped off and spun down at 14,000 rpm (4 °C for 15 min). Supernatants were then stored at −80 °C. High throughput kinase activity mapping (HT-KAM) is a platform using arrays of peptides that act as sensors of phosphorylation activity^28^. The phospho-catalytic signature of samples was measured in the presence of individual peptides that are experimentally isolated from each other, and established from simultaneously occurring ATP-consumption tests. HT-KAM assays were run in 384 well-plates and each experimental well contained one peptide. The final 8 µL reaction mixtures per well contained: (1) kinase assay buffer (1X KaB: 2.5 mM Tris-HCl (pH7.5), 1 mM MgCl_2_, 0.01 mM Na_3_VO_4_, 0.5 mM glycerophosphate, 0.2 mM dithiothreitol (DTT), prepared daily; (10X KaB Cell Signaling Technology®, cat.no.: 9802), (2) 250 nM ATP (prepared daily with 1X KaB; Cell Signaling Technology® cat.no.: 9804), (3) 200 µg/ml 11-mer peptide (lyophilized stocks originally prepared as 1 mg/ml in 1X KaB, 5% DMSO), as well as (4) samples made from cells at ~10 µg/ml total protein extract. Before being used, samples were kept on ice and diluted in 1X KaB <30 min. Samples were run side-by-side within each 384 well-plate including controls with no-ATP, or no-peptide, or no-sample as well as ATP standards. A Biomek® FX Laboratory Automation Workstation from Beckman Coulter was used for high-throughput liquid dispensing of all reagents. Reagents were kept on ice at all times and plates on cold blocks until enzymatic reactions started. After the dispensing of the reaction mixtures, the plates were incubated for 1 h at 30 °C. A kinase-Glo revealing reagent (Promega®; cat.no.: V3772) was used for ATP detection, which stops the activity of the kinases and produces a luminescent signal that directly correlates with the amount of remaining ATP in the samples. Luminescence was measured using the Synergy 2 Multi-Mode Microplate Reader from BioTek, and luminescence data were inversely correlated with the amount of kinase activity. A more detailed description of the peptide sensors design, sequence and connectivity between peptides and kinases, as well as data normalization steps and analysis, can be found in these publications^28–30^. The activity of kinase enzymes was sourced from their respective subset of biological peptide targets included in the assay.

### Dual treatment synergy analysis

The responses to drug combinations on cell growth were analyzed using the highest single agent (HSA, or Gaddum’s non-interaction model) model, which represents the idea that synergistic drug combinations produce additional benefits on top of what its components can achieve alone. HSA scores were obtained, using the SynergyFinder+ web application^31^. For dual treatment regimen the inhibitors trametinib (Selleck Chemicals, cat.no.: S2673), palbociclib (Selleck Chemicals, cat.no.: S1116), pemigatinib (TargetMol® cat.no.: T12401), and Selpercatinib (MCE® cat.no.: HY-114370) were used. Cells were treated for three or five days.

### Statistics and reproducibility

Student’s t-test was used for *p*-value calculations (significance was defined for *p* < 0.05). Statistical tests were calculated using software (Microsoft® Excel Version 2107).

### Reporting summary

Further information on research design is available in the Nature Portfolio Reporting Summary linked to this article.

## Results

### *NRAS*-mRNA is a targetable and strongly selective vulnerability in *NRAS*-mutant melanoma

**Fig. 1:**
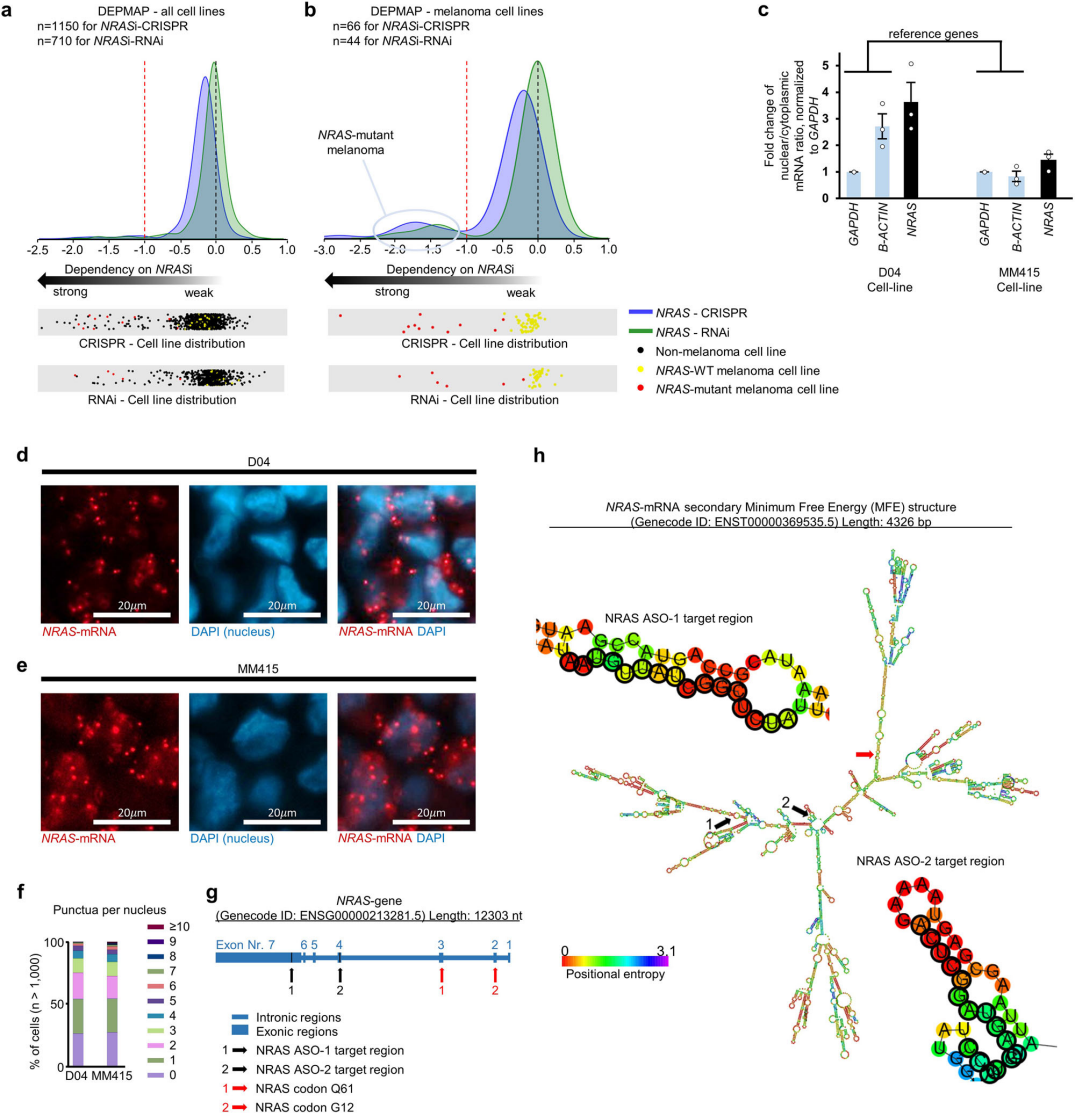
*NRAS*-mRNA is a targetable and strongly selective vulnerability in *NRAS*-mutant melanoma. **a** Analysis of response of cell lines from the Dependency Map portal (DepMap) database to CRISPR-knockout (blue curve) or RNAi-mediated inhibition of *NRAS*-mRNA (green curve) shows that the vast majority of cell lines presented no dependency on *NRAS*-mRNA expression (dependency score 0, black dotted line). **b** Filtering for melanoma cell lines showed that specifically *NRAS*-mutant melanoma cells presented a strong vulnerability on *NRAS*-mRNA expression (dependency score ≤ -1, red dotted line). Dot plots represent all analyzed cell lines (black: non-melanoma, yellow: *NRAS* wild type melanoma, red: *NRAS*-mutant melanoma), highlighting that the dependent melanoma cell lines harbor *NRAS* mutations. **c** Subcellular mRNA enrichment analysis was done using qRT-PCR to compare the ratio of nuclear versus cytoplasmic mRNA levels of *NRAS*, *GAPDH* and *B-ACTIN* in D04 and MM415 cells. The data are presented as fold-change of nuclear to cytoplasmic ratio normalized to *GAPDH* (*n* = 3) and show higher nuclear enrichment of *NRAS-mRNA*, when compared to reference genes. The error bars represent Standard Error (s.e.m.). **d**, **e** Representative images of RNA in situ hybridization (RNA-ISH) derived from **d** D04 and **e** MM415 cell pellets. Fluorescent signals are either produced by DAPI DNA staining to mark the nuclear regions (blue) or probes that stain the *NRAS*-mRNA (red). **f** Quantification of punctua per nucleus from fluorescent signals produced by probes that stain *NRAS*-mRNA in D04 and MM415 cells. The calculations included > 1000 cells per cell line. **g** Intronic (small bars) and exonic (large bars) regions of the *NRAS* gene (ENSG00000213281.5) as annotated in the Genecode database (V44). *NRAS* ASO target regions are highlighted in black and the codons Q61 and G12 are highlighted in red. **h** *NRAS*-mRNA (Genecode ID: ENST00000369535.5) secondary structure as predicted by the Minimum Free Energy (MFE) model. NRAS ASO target regions are highlighted in black, provided in additional cutout and zoom. Codon Q61 is highlighted in red. The ASO target regions represent stable and accessible structures.

The mutation status of *RAS*-oncogenes has been proven to serve as an important biomarker of cancer cells’ responsiveness to certain anti-cancer therapies^32,33^. To analyze whether the mutation status of the *NRAS* oncogene can serve as biomarker for responsiveness to *NRAS*-mRNA targeting therapy, we analyzed *NRAS*-mRNA knockdown effects in human cell lines, using the Cancer Dependency Map portal (https://depmap.org/portal/). This analysis involved data for responses to reduced *NRAS*-mRNA levels of more than one thousand cell lines, covering over 30 different cancer types (specifically CRISPR mediated loss-of-function data from 1150 cell lines and RNAi data from 710 cell lines). Cell lines that presented an inhibition effect of ≤ −1 were considered as having a strong dependency to *NRAS*-mRNA expression (dashed red line in Fig. 1a, b, see methods for details about the definition of the dependency score). NRAS-dependency was only observed in a low number of cell lines. Altogether, 48 cell lines (4.2%) of the CRISPR knockdown group and 16 cell lines (2.3%) of the RNAi knockdown group showed a strong dependency to *NRAS*-mRNA expression (Fig. 1a, Supplementary Data 1). However, a large fraction of the NRAS-dependent cell lines harbored *NRAS*-mutations (>85% in the CRISPR, and >94% in the RNAi group). Next, we investigated NRAS-dependency in the subgroup of all melanoma cell lines. We sorted the cell lines for their *NRAS*-mutation status and identified that 0% of *NRAS*-WT melanoma cell lines showed strong dependency on *NRAS*-mRNA expression. (Fig. 1b, Supplementary Data 1; 0 of 55 *NRAS*-WT melanoma cell lines in the CRISPR-group and 0 of 38 *NRAS*-WT melanoma cell lines in the RNAi-group). In contrast, a strong dependency on *NRAS*-mRNA expression was observed in 81.8% of *NRAS*-mutant melanoma cell lines (9 of 11) in the CRISPR group and in 66.7% of *NRAS*-mutant melanoma cell lines (4 of 6) in the RNAi group (Fig. 1b, Supplementary Data 1). Strong dependencies to *NRAS*-mRNA expression were also observed in multiple NRAS-mutant cancer cell lines of non-melanoma cancers (Fig. 1a, Supplementary Data 1; i.e., Neuroblastoma, Acute Myeloid Leukemia, Ovarian cancers, and Small Cell Lung Cancer).

To investigate the cellular distribution of *NRAS*-mRNA, we conducted subcellular fractionation analysis in two *NRAS*-mutant melanoma cell lines (D04 and MM415). *NRAS*-mRNA was identified in the nucleus and cytoplasm in both cell lines and showed equal to stronger nuclear enrichment when compared to the mRNA of the reference genes *GAPDH* and *Β-ACTIN* (Fig. 1c; when compared to the reference genes, the nuclear enrichment of *NRAS*-mRNA was 2-fold higher in D04 and 1.6-fold higher in MM415, when normalized to *GAPDH*). The distribution of *NRAS*-mRNA across nuclear and cytoplasmic compartments was further confirmed in both cell lines using RNA in situ hybridization (RNA-ISH) on formalin fixed and paraffin embedded D04 and MM415 cell pellets (Fig. 1d, e). Quantification of RNA-ISH staining derived *NRAS*-mRNA signals (>1000 cells per cell line) showed similar distribution patterns of fluorescent punctua per nucleus in the D04 and MM415 cell lines (Fig. 1f).

Based on these findings, we aimed to identify a treatment strategy that can be used to efficiently target and deplete *NRAS*-mRNA and holds the potential to be translated into clinical settings. We chose to pursue additional RNA-targeting experiments with ASOs, which are commonly used for depleting RNAs in pre-clinical research, as well as in clinical trials and in FDA and EMA approved treatments of various diseases^19,34^.ASOs are active in both the nucleus and cytoplasm^35^. The *NRAS* gene (Genecode ID: ENSG00000213281.5, GENCODE project, V38) is located on the reverse strand of Chromosome 1 and is transcribed as a single isoform (Fig. 1g represents a schematic illustration of the genomic regions of the *NRAS* gene, the ASO targeting sites, codon Q61, which is the most frequently affected location for MAPK-pathway inducing *NRAS*-mutations, and codon G12, which is another potential location for NRAS-driving mutations addressed in this study). We designed two GapmeR ASOs that include a 16-nucleotide long *NRAS*-mRNA targeting sequence, with NRAS ASO-1 targeting Exon 7 and NRAS ASO-2 targeting Exon 4 (Fig. 1g, Supplementary Table 1). The binding affinity of ASOs to their target mRNA can be influenced by various factors, including the chemical modifications of the ASOs, and the target region accessibility of the mRNA^36^. The positional entropy of the targeted nucleotides can play a crucial role in assessing accessibility for effective ASO binding. A low value of positional entropy may indicate a higher probability of nucleotides to stay in the same configuration, therefore providing stable accessibility^37^, while instable ASO:mRNA complexes may hinder RNaseH activity and therefore ASO efficiency^38^. A computational illustration of the Minimum Free Energy (MFE) secondary structure of the *NRAS*-mRNA shows that the NRAS ASO target sites exclude regions of high positional entropy (Fig. 1h, Supplementary Fig. 1a, b). The specificity of an ASO construct is reinforced by its inability to bind to non-target RNAs. The effectiveness of GapmeR ASOs substantially diminishes with a single mismatch to a target region, and the presence of two mismatches results in the complete deactivation of the ASO^39^. To account for potential off-target binding, we matched the ASO sequences to the human transcriptome. Both NRAS ASOs exclusively target the *NRAS*-mRNA with 100% specificity. The identified top20 off-targets, ranked by alignment expectation value (e-value) that bear the closest resemblance to the NRAS target sequences, include at least 3 mismatches for NRAS ASO-1 and 4 mismatches for NRAS ASO-2 (Supplementary Table 2+3). In summary, these findings highlight that the designs of NRAS ASO-1 and NRAS ASO-2 meet the requirements to target *NRAS*-mRNAs efficiently and specifically.

### NRAS ASO treatment reduced *NRAS*-mRNA, NRAS-protein levels, and MAPK-pathway signaling

**Fig. 2:**
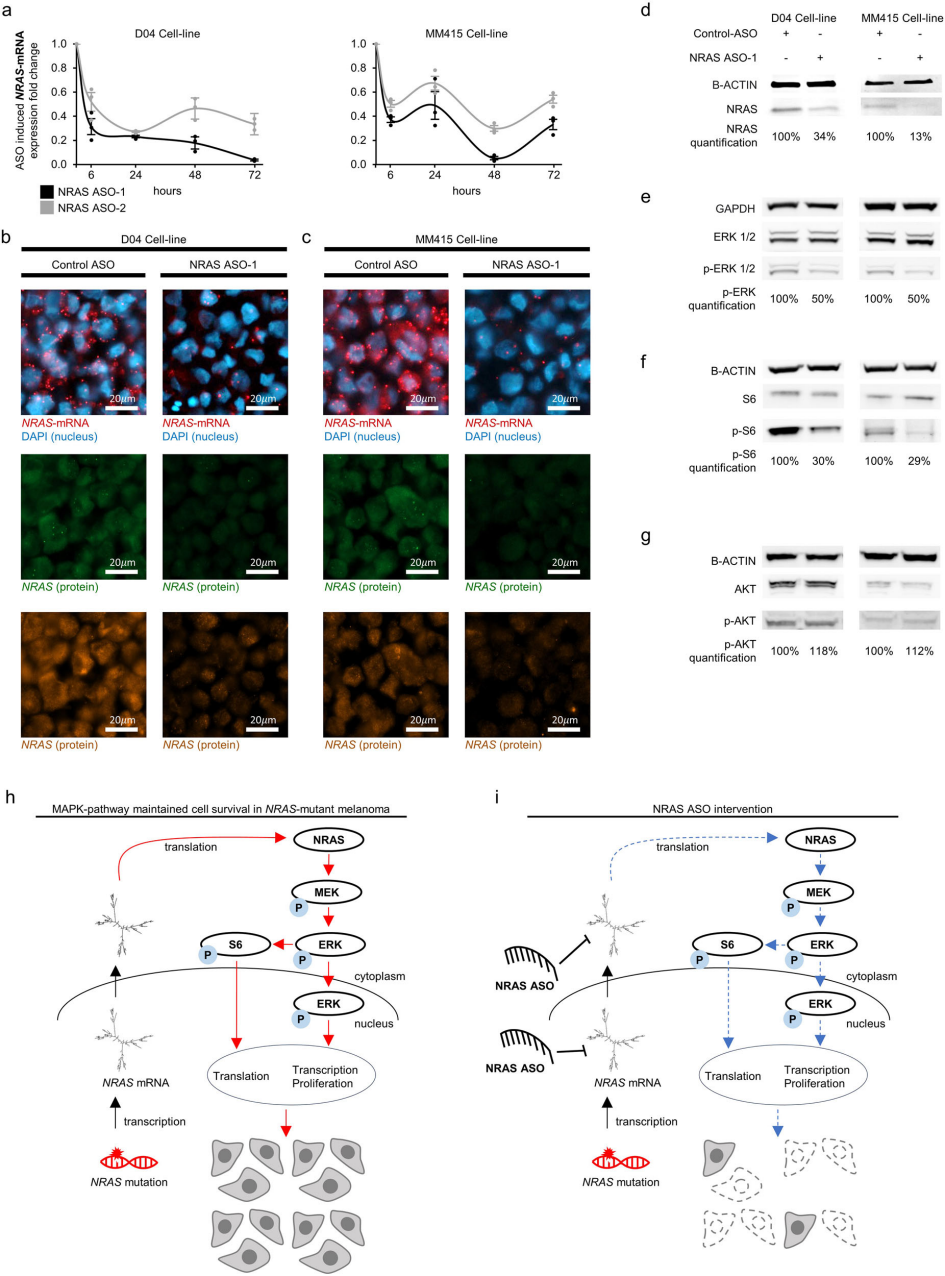
NRAS ASO treatment reduces *NRAS*-mRNA, protein levels, and MAPK-pathway signaling in *NRAS*-mutant melanoma. **a** Using qRT-PCR to compare RNA levels in D04 and MM415 cells that were either treated with NRAS ASO-1 or NRAS ASO-2, showed a robust reduction of *NRAS*-mRNA levels after 6, 24, 48, and 72 hours, when compared to treatment with non-targeting Control ASO. Final oligonucleotide concentration was 100 nM; error bars represent s.e.m. (*n* = 3). **b**, **c** Representative images of RNA in situ hybridization (RNA-ISH) derived from pellets of **b** D04 or **c** MM415 cells, either treated with NRAS ASO-1, or Control ASO. Fluorescent signals were produced by DAPI DNA staining to mark the nuclear regions (blue), probes that stain the *NRAS*-mRNA (red), and two different antibodies that stain for NRAS protein (ProteinTech 10724-1-AP – green, LsBio LS-C174539 – orange). NRAS ASO-1 treatment strongly reduced *NRAS*-mRNA levels in the cytoplasm and nucleus of the cells and NRAS protein expression. Final oligonucleotide concentration was 100 nM and treatment period lasted for 24 h. **d** Immunoblotting showing a strong decrease in NRAS protein levels 1 day after NRAS ASO-1 treatment compared to Control ASO treatment in D04 (−66%) and MM415 (−87%) cell lysates. B-ACTIN served as loading control and normalization parameter. **e** Immunoblotting showing a decrease in p-ERK1/2 protein levels 2 days after NRAS ASO treatment compared to Control ASO treatment in D04 (−50%) and MM415 (−50%) cell lysates, while total ERK1/2 levels were not altered significantly. GAPDH served as loading control and normalization parameter. **f** Immunoblotting showing a decrease in p-S6 protein levels 2 days after NRAS ASO-1 treatment compared to Control ASO treatment in D04 (−70%) and MM415 (−71%) cell lysates, while total S6 levels were not altered significantly. **g** Immunoblotting showing a small increase in p-AKT protein levels 2 days after NRAS ASO-1 treatment compared to Control ASO treatment in D04 (+18%) and MM415 (+12%) cell lysates. Total AKT levels were not altered significantly. Final oligonucleotide concentration was 100 nM. **h** A simplified illustration depicting key signaling pathways in *NRAS*-mutant melanoma, emphasizing the activation of crucial proteins contributing to cellular survival. Through transcription, the mutations in the *NRAS* gene are carried over to the *NRAS*-mRNA, which is translated into the constitutively active mutant NRAS protein, initiating downstream signaling cascades. This activation prompts the RAF kinase (not shown) to activate MEK, which, in turn, activates ERK. ERK signaling influences the activation of S6 ribosomal protein and translocates to the nucleus, regulating transcription and supporting cellular proliferation. S6 plays a pivotal role in translation, facilitating protein synthesis. The activation of this signaling pathways enhances cellular survival in *NRAS*-mutant melanoma. Phosphorylation-dependent activation steps are denoted by (P). **i** A simplified illustration highlighting the impact of NRAS ASO treatment: NRAS ASOs reduce *NRAS*-mRNA levels in both the cytoplasm and nucleus. This reduction is followed by decreased NRAS protein levels and the inhibition of MAPK-pathway signaling activity, as evidenced by diminished p-ERK and p-S6 protein levels. The pathway is essential for the NRAS-mutant cancer cells’ ability to proliferate and survive.

The efficacy of NRAS ASO-1 and NRAS ASO-2 was assessed in two *NRAS*-mutant melanoma cell lines at four time points, showing that the treatment can reduce *NRAS*-mRNA levels by up to 95%, with a peak depletion observed between 48 and 72 h. (Fig. 2a, *NRAS*-mRNA expression in D04 and MM415 cells at 6, 24, 48 and 72 hours after ASO incubation). NRAS ASO-1 consistently outperformed NRAS ASO-2 across all time points in both cell lines. Reduced *NRAS*-mRNA and protein levels upon NRAS ASO-1 treatment were observed using RNAscope and immunofluorescence staining of formalin-fixed and paraffin-embedded (FFPE) D04 and MM415 cell pellets (Fig. 2b, c). The reduction of NRAS protein levels was evaluated by immunofluorescence using two different NRAS-targeting antibodies (Fig. 2b, c). Quantification of protein levels detected by immunoblot confirmed that NRAS ASO-1 treatment caused a strong reduction of NRAS protein levels (Fig. 2d, 66% reduction in D04 cells and up to 87% reduction in MM415). The NRAS ASO-1 mediated inhibition of MAPK-signaling downstream of NRAS was confirmed by immunoblot. NRAS ASO-1 treated D04 and MM415 cells showed reduced protein levels of the activated signaling kinases p-ERK1/2, which are located downstream in the MAPK-pathway cascade (Fig. 2e)^40^. The ribosomal protein S6 is another mediator of MAPK-signaling, its activation occurs downstream of p-ERK1/2 signaling within the MAPK pathway cascade^41^. NRAS-ASO-1 treatment subsequently reduced the levels of activated ribosomal protein S6 (Fig. 2f, p-S6). Previous studies highlighted interactions between the MAPK and PI3K/AKT signaling pathways in melanoma, sharing closely connected co-regulatory mechanisms that are essential for cellular survival^42^. Immunoblot analysis showed that NRAS ASO-1 did not substantially affect p-AKT levels (Fig. 2g), highlighting the specific inhibitory effect of ASO-mediated *NRAS*-mRNA depletion on the MAPK-signaling axis in NRAS-mutant melanoma. Total protein levels of ERK1/2, S6, and AKT were not considerably affected by the treatment (Fig. 2e–g). We investigated the potential effects NRAS ASO-1 may have on the mRNA expression of other RAS gene family members 24 hours after treatment with NRAS ASO-1. This is a time point at which we observed significant reduction in *NRAS*-mRNA levels and protein expression (Fig. 2a–d). We found that NRAS ASO-1 treatment led to a modest upregulation of *HRAS*-mRNA (D04: 1.27-fold, SEM = 0.12; MM415: 1.33-fold, SEM = 0.14) and caused minimal changes in *KRAS*-mRNA expression (D04: 1.08-fold, SEM = 0.05; MM415: 0.83-fold, SEM = 0.11). These results indicate that the reduction in *NRAS*-mRNA and protein levels has minimal to no impact on *HRAS* or *KRAS*-mRNA expression (Supplementary Fig. 1c).

Figure 2h illustrates the MAPK pathway-related mechanisms facilitating sustained cell survival in *NRAS*-mutant melanoma, while Fig. 2i illustrates the antiproliferative effects of NRAS ASO treatment, and inhibition of the MAPK signaling stream.

### NRAS ASO treatment significantly induced apoptosis and reduced cell and tumor growth in *NRAS*-mutant melanoma

**Fig. 3:**
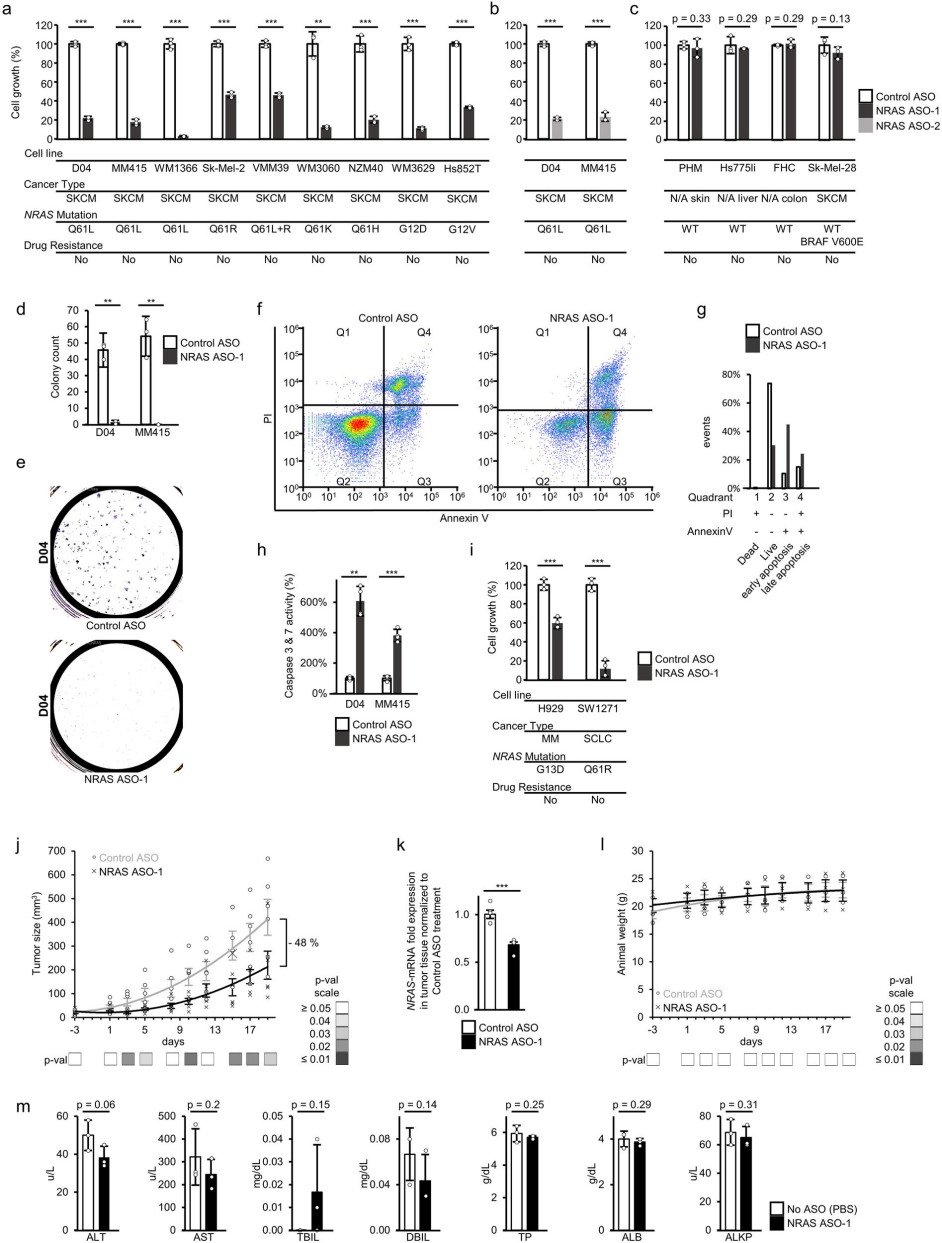
NRAS ASO treatment significantly induces apoptosis and specifically reduces cell and tumor growth in *NRAS*-mutant melanoma. **a** Treatment with NRAS ASO-1 caused significant inhibition of cell growth in the *NRAS*-mutant melanoma cell lines D04 (*p* = 0.000002), MM415 (*p* = 0.00002), WM1366 (*p* = 0.0005), Sk-Mel-2 (*p* = 0.00001), VMM39 (*p* = 0.00004), WM3060 (*p* = 0.003), NZM40 (*p* = 0.0006), WM3629 (*p* = 0.0008), and the primary derived cell line Hs852T (*p* = 0.000006). **b** Treatment with NRAS ASO-2 caused significant inhibition of cell growth in the *NRAS*-mutant melanoma cell lines D04 (*p* = 0.000004) and MM415 (*p* = 0.0001). The antiproliferative outcomes are similar when compared to treatment with NRAS ASO-1. **c** NRAS ASO treatment did not cause significant antiproliferative effects in primary human melanocytes (PHM, *p* = 0.33), primary human liver cells (Hs775li, *p* = 0.29), human colon cells (FHC, *p* = 0.29), and BRAF-mutant melanoma cells (Sk-Mel-28, *p* = 0.13). **d** NRAS ASO treatment significantly inhibited colony formation in the D04 (*p* = 0.0017) and MM415 (*p* = 0.008) cell lines compared to treatment with non-targeting Control ASOs. Treatment period was 7 days (50 nM final oligonucleotide concentration, *n* = 3). **e** Representative images of D04 colonies in 6 cm dishes after ASO treatment. **f** Dot plot graph of flow cytometric analysis of PI and Annexin V staining after 1 day of ASO-treatment (100 nM) shows increased apoptotic cell death in D04-cells treated with NRAS ASO (15,780 total events) compared to Control ASO treatment (44,285 total events). **g** Distribution of overall cell populations from panel **f**) in regards of their apoptotic state. Bars represent the percentage of vital (Q2), early apoptotic (Q3), late apoptotic (Q4) and dead (Q1) cells. **h** NRAS ASO-mediated induction of apoptosis was confirmed by measurement of significantly increased activity levels of the apoptosis markers Caspase-3 & -7 after 1 day of treatment with either NRAS or Control ASOs (100 nM) in the D04 (*p* = 0.002) and MM415 (*p* = 0.0002) cell lines (*n* = 4). **i** Treatment with NRAS ASO−1 caused significant inhibition of cell growth in the *NRAS*-mutant multiple myeloma (MM) cell line H929 (*p* = 0.0005), and small cell lung cancer (SCLC) cell line SW1271 (*p* = 0.0001). **j** Significant tumor growth reduction was observed when comparing treatment groups for subcutaneous systemic treatment with either NRAS ASO (X) or Control ASO (O) in mouse models carrying xenografts of the D04 melanoma cell line (3 × 200 µg ASO/week, *n* = 6, days of measurement and *p*-values: −3 –0.38, 1 –0.27, 3 –0.02, 5 –0.04, 8 –0.05, 10 – 0.02, 12 –0.06, 15 –0.02, 17 – 0.02, 19 – 0.03). At the endpoint of the experiment (day 19), the average tumor size in the NRAS ASO treatment group was 48% smaller compared to control. **k** *NRAS*-mRNA levels were significantly reduced (0.68-fold, s.e.m = 0.03, *p* = 0.0003) in tumors of the NRAS ASO treatment group compared to the Control ASO treatment group at the end of study period. Tumors were harvested at end of treatment period; gene expression was normalized to *Β-ACTIN* expression and *NRAS*-mRNA expression in NRAS ASO treated tumors was normalized to expression in Control ASO treated tumors (*n* of each group = 5). **l** No significant weight changes were observed between the NRAS ASO (X) and Control ASO (O) groups at any time-point (days of measurement and *p*-values: -3 – 0.3, 1 – 0.36, 3 – 0.33, 5 – 0.46, 8 – 0.43, 10 – 0.5, 12 – 0.47, 15 – 0.49, 17 – 0.48, 19 – 0.49). **m** Blood of mice that either received a dose of NRAS ASO-1 (200 µg/injection), or ASO-free PBS was drawn 24 hours after injection and analyzed for parameters of liver function (Serum transaminases – ALT, AST, bilirubin - TBIL, direct (conjugated) bilirubin - DBIL, total protein - TP, albumin - ALB, and alkaline phosphatase – ALKP). All growth and weight curves are presented as polynomial trend lines (order: 2). Data in (**a**–**c**, **i**) were normalized to treatment with non-targeting Control ASO, final oligonucleotide concentration was 50 nM, treatment period was 5 days (*n* = 3). The error bars in **a**–**d**), **h**, **i**, **m**) represent s.d., in **j**−**l** they represent s.e.m. Significance is shown as *p*-values calculated by Student’s t-test. * =*p* < 0.05, ** =*p* < 0.01, *** =*p* < 0.001.

We tested the impact of NRAS ASO-1 treatment on cell proliferation in nine *NRAS*-mutant melanoma cell lines, including the primary derived cell line Hs852T (Fig. 3a). The treatment caused strong and significant reduction of cell growth, when compared to non-targeting Control ASO treatment (Fig. 3a). The impact of NRAS ASO-1 treatment on cell growth varied in intensity across different cell lines. These differences may be attributed to variations in ASO internalization or mRNA-depletion between cell lines, as illustrated by comparison of D04, MM415 and VMM39 cells. VMM39 presented reduced NRAS ASO mediated inhibition of cell growth when compared to D04 and MM415 (Fig. 3a, VMM39: −54%, D04: −78%, MM415: −82%). These observations aligned with reduced NRAS ASO-1 mediated reduction of *NRAS*-mRNA levels (Fig. 2a**+** Supplementary Fig. 1d, VMM39: −33%, D04: −69%, MM415: −64% after 6 h of NRAS ASO-1 treatment).

To assess potential non-specific toxicity of NRAS ASO treatment, we evaluated the cell growth-reducing effects of two additional components used in our investigation of NRAS ASOs: Control ASO treatment, and the transfection reagent Lipofectamine, which was used for all in vitro ASO transfection experiments in this study. The control ASO sequence does not target any transcripts in the human transcriptome (Supplementary Table 4), making it a suitable tool for assessing non-specific effects unrelated to RNase H-mediated target degradation. Compared to untreated cells, control ASO treatment resulted in a minor but significant reduction in cell growth in both the D04 (Supplementary Fig. 1e, −8.6 %, *p* = 0.01), and MM415 (Supplementary Fig. 1f, −13.6%, *p* = 0.002) cell lines. However, these effects are likely attributable to the transfection reagent, as no significant differences in cell growth were observed when comparing cells treated with the transfection reagent alone (excluding ASO) to those treated with control ASO and transfection reagent (Suppl. Figure 1e, f, D04: *p* = 0.17, MM415: *p* = 0.11).

Next, we compared the cell growth-inhibiting effects of NRAS ASO treatment to NRAS siRNA treatment. Although we used an optimized siRNA design, which enhances efficiency and reduces off-target interactions by pooling siRNAs that target different sites on NRAS^43^, the ASO treatment outperformed the siRNA treatment in one out of two cell lines. In the D04 cell line, NRAS siRNA treatment significantly inhibited cell growth compared to control ASO (−69%), and in a direct comparison, the siRNA treatment caused significantly less cell growth inhibition than NRAS ASO-1 (Fig. 3a**+** Supplementary Fig. 1g, −69 % VS −78 %, *p* = 0.01). In MM415 cells, the siRNA treatment also significantly inhibited cell growth, with no significant difference compared to NRAS ASO-1 treatment (Fig. 3a**+** Supplementary Fig. 1h, −82 % VS −82 %, *p* = 0.46).

Then, we compared the cell growth-inhibiting effects of NRAS ASO-1 + 2 treatment to treatment with an NRAS-targeting ASO that is specifically directed to the mRNA sequence harboring the NRAS^Q61L^ mutational site (NRAS ASO-Q61L) in the NRAS^Q61L^ mutated melanoma cell lines D04 and MM415. NRAS ASO-1 + 2 demonstrated superior efficiency compared to NRAS ASO-Q61L (Fig. 3a**+** Supplementary Fig. 1i,j, Supplementary Table 5). The specificity of the NRAS ASOs was further assessed using single-nucleotide mismatch versions of NRAS ASO-1 (NRAS ASO-1 MM) and NRAS ASO-2 (NRAS ASO-2 MM). These single-nucleotide mismatches caused significantly less cell growth inhibition compared to their original counterparts (Supplementary Fig. 2a, b, Supplementary Table 6+**7**). The structure and chemical modifications of all NRAS-targeting antisense constructs used in this study is listed in Supplementary Table 1.

To further address whether the observed NRAS ASO-1 mediated cell-growth inhibition is linked to the depletion of *NRAS*-mRNA levels, we treated D04 and MM415 cells with NRAS ASO-2, producing highly similar effects (Fig. 3a, b).

Building on the findings shown in Fig. 1a, b, which indicated that *NRAS*-mRNA expression may be a specific vulnerability of *NRAS*-mutant melanoma cells, we applied the treatment to three normal, non-cancerous cell lines, and a BRAF^V600E^-mutant melanoma cell line, to evaluate whether NRAS-ASOs provoked toxic side effects in *NRAS*-WT cell lines. NRAS ASO-1 treatment did not significantly reduce cell growth in primary human melanocytes (the cell type that melanoma derives from), primary human liver cells, epithelial human colon cells, or NRAS-WT melanoma cells (Fig. 3c). To test the effect of NRAS ASO treatment on colony formation, we applied either NRAS ASO-1 or Control ASO treatment in clonogenic assays of D04 and MM415 cells. NRAS ASO-1 treatment significantly reduced the cells’ capability to form colonies (Fig. 3d, e). Notably, colony formation was completely inhibited in the MM415 melanoma cell line (Fig. 3d, “missing” bar graph on the right).

To investigate the nature of NRAS ASO induced cell death, we treated D04 cells with either NRAS or Control ASOs, stained them with Annexin-V and propidium iodide (PI), and performed flow cytometry to differentiate live, dead, and apoptotic cells (Fig. 3f, g). After 1 day of treatment, NRAS ASO-1 caused a decrease of healthy cells (30% vs 74%) and a strong increase of cells in early (45% vs 11%) and late apoptosis (Fig. 3f, g, 24% vs 15%). To further confirm the apoptosis inducing effect triggered by NRAS ASOs, we measured activity of the apoptosis executioner caspases-3 & −7 of cells treated with either NRAS ASO-1 or Control ASOs. NRAS ASO-1 increased caspase activity significantly and strongly in the D04 and MM415 cell lines (Fig. 3h, D04: > 600%, *p* = 0.002; MM415: > 380%, *p* = 0.0002).

To test, whether the specific vulnerability of *NRAS*-mutant cancer cells to NRAS inhibition expands beyond melanoma, we tested if the treatment may impact cell-growth in *NRAS*-mutant multiple myeloma (MM, H929) and small cell lung cancer (SCLC, SW1271) cells. The treatment with NRAS ASO-1 significantly reduced cell growth in both *NRAS*-mutant cell lines (Fig. 3i). Some cell lines that were tested for NRAS ASO treatment, were also included in the DepMap dataset for NRAS dependency analysis. (Fig. 3a, c+i, Supplementary Data 1, Sk-Mel-2 - CRISPR: −2.8, RNAi: −1.4; Hs852T - CRISPR: −0.6, RNAi: −0.2; Sk-Mel-28 - CRISPR: n/a, RNAi: −0.06; and SW1271 - CRISPR: −1,8, RNAi: −1.9).

To extrapolate the implications of our findings and to further evaluate the potential clinical significance of *NRAS* ASO intervention, we conducted an in vivo study in mouse models carrying melanoma D04 cell line-derived xenografts. We administered subcutaneous ASO injections (200 µg/injection) three times a week (600 µg/week), over the course of three weeks. Our observations indicated a significant reduction of the average tumor size within the NRAS ASO-1 treatment group compared to the Control ASO group (Fig. 3j). Notably, on day three of the in vivo experiment timeline, the tumor size of mice that received NRAS ASO-1 was already significantly smaller compared to the Control ASO treatment group (20  mm^3^ vs 64 mm^3^, *p* = 0.02). At this timepoint, the mice had only received a single dose of systemic ASO treatment. The trend continued and at the planned endpoint of the experiment the tumors in the NRAS ASO-1 treatment group were still significantly smaller compared to the Control ASO treatment group (−48 %, 220 mm^3^ vs 422 mm^3^, *p* = 0.03). When matched to the mouse transcriptome, the top 5 hits ranked by e-value for the NRAS ASO-1 sequence present at least 3 mismatches to potential off-targets, indicating a low likelihood of off-target related toxicity (Supplementary Table 8). Tumors that were treated with NRAS ASO-1 presented reduced *NRAS*-mRNA levels (Fig. 3k, 0.68-fold), when compared to tumors of mice that were treated with Control ASOs. To address unspecific potential toxic side effects caused by in vivo systemic application of NRAS ASO-1, we measured mouse weight over the course of the study period, which remained stable and did not show significant differences among the study groups at any time point of measurement (Fig. 3l). To evaluate potential hepatotoxic side effects of the ASO treatment, blood samples from mice were analyzed for liver function parameters 24 h after receiving either a single NRAS ASO-1 (200 µg/injection), or an ASO-free PBS injection. The NRAS ASO-1 treatment group presented lowered serum transaminase levels compared to the control group, the differences were not significant (Fig. 3m, ALT: 50 u/L vs 38.3 u/L, p = 0.06; AST: 244 u/L vs 321.3 u/L, *p* = 0.2). Additionally, no significant differences could be measured for the liver function parameters bilirubin, direct (conjugated) bilirubin, total protein, albumin, and alkaline phosphatase (Fig. 3m, TBIL *p* = 0.15, DBIL *p* = 0.14, TP *p* = 0.25, ALB *p* = 0.29, and ALKP *p* = 0.31). All mice underwent continuous monitoring for activity levels and signs of distress or pain. No discernible differences were observed between the treatment groups during this monitoring period.

### The regulatory role of NRAS in kinase activity signatures reveals dual treatment regimen with synergistic potential

**Fig. 4:**
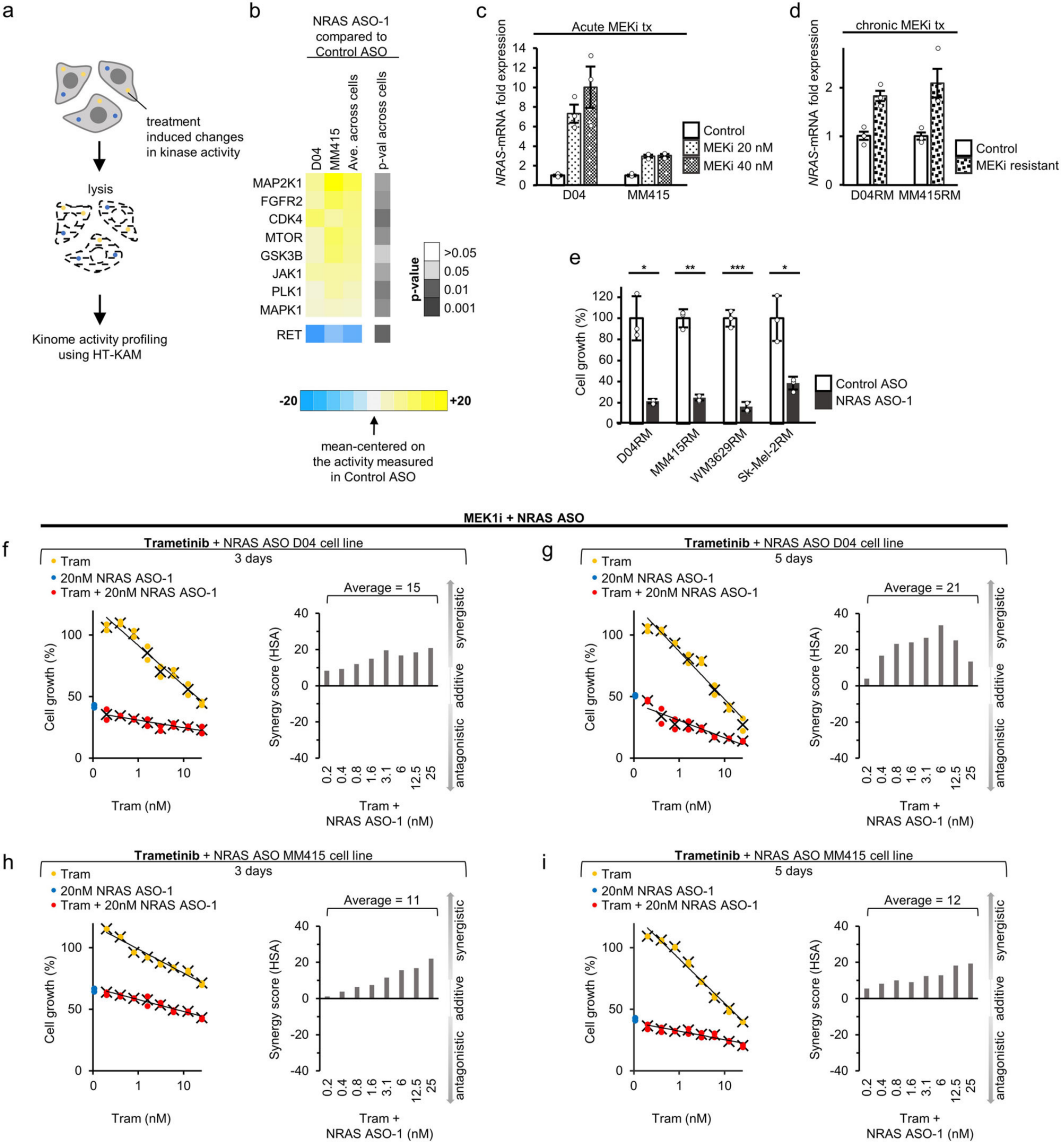
The regulatory role of NRAS in kinase activity signatures reveals dual treatment regimen with synergistic potential. **a** Schematic illustration of HT-KAM analysis of the phosphor-catalytic activity of kinases. D04 and MM415 cells were either treated with NRAS or Control ASOs (50 nM, 1 day). Cells were lysed, and protein lysate was investigated for peptide-associated phosphorylation activity of kinases. **b** Comparison of kinase activity in treatment groups (NRAS ASO VS. Control ASO) showed that kinase activity of several kinases was significantly upregulated in the D04 and MM415 cell lines upon NRAS ASO treatment. Kinases are ranked by their relative increase of activity (from bottom to top). The top 3 kinases with strongest shift in activity increase are MAP2K1 (MEK1), FGFR2, and CDK4. The RET kinase activity shift is shown as a representative example for kinases that were downregulated in activity. **c** QRT-PCR analysis showing elevated *NRAS*-mRNA levels in D04 and MM415 cells after three days of drug-induced Inhibition of MEK (MEKi), using the small molecule inhibitor Trametinib (20 nM or 40 nM), when compared to control, treated with DMSO (*n* = 3). **d** QRT-PCR analysis showing elevated *NRAS*-mRNA levels in the MEKi resistant cell lines D04RM and MM415RM, which were chronically exposed to Trametinib, when compared to their paternal treatment naïve cell lines D04 and MM415 (*n* = 3). Error bars in panel (**c**, **d**) represent s.e.m. **e** Treatment with NRAS ASO-1 caused significant inhibition of cell growth in the MEKi resistant *NRAS* mutant melanoma cell lines D04RM (p = 0.011), MM415RM (*p* = 0.001), WM3629RM (*p* = 0.0002), and Sk-Mel-2RM (*p* = 0.015). Data were normalized to treatment with non-targeting Control ASO; treatment period was 5 days, final oligonucleotide concentration was 50 nM, and error bars represent s.d. (*n* = 3). **f**–**i** Dual treatment with 20 nM of NRAS ASO and Trametinib (Tram, 0.5 nM −25 nM) caused robust synergistic effects in D04 (**f**, **g**) and MM415 (**h**, **i**) cells after 3 (**f**, **h**) and 5 (**g**, **i**) days of treatment (*n* = 2). Dose response curves show NRAS ASO treatment (blue), trametinib treatment (yellow) and dual treatment (red). Synergism of dual cell growth inhibition is shown as bar graphs and determined by the HSA synergy score.

Drugs that target MAPK-pathway signaling offer limited clinical benefit for *NRAS*-mutant melanoma patients due to early onset treatment resistance^3^. Such resistance mechanisms may arise as cancer cells restore pro-survival signaling through the activation of alternative kinase signaling pathways^3^. We used a high-throughput kinase activity mapping assay (HT-KAM)^28–30,44^, to identify potential kinases whose activity is increased upon NRAS ASO-1 treatment (Fig. 4a provides a schematic illustration of the workflow to identify the cells’ phospho-catalytic fingerprints; see Methods for assay details). HT-KAM analysis of the phospho-fingerprint of D04 and MM415 cells unveiled that the activity of several kinases was significantly upregulated in NRAS ASO-1 treated cells in comparison to Control ASO, with MAP2K1 (*p* = 0.032), FGFR2 (*p* = 0.028) and CDK4 (*p* = 0.017) kinases displaying the highest increase (Fig. 4b).

To assess the potential therapeutic value and druggable susceptibility, we initially focused on the upregulation of MAP2K1 (*MEK*1) upon NRAS ASO-1 treatment. Targeting the MAP2K kinases in *NRAS*-mutant melanoma represents a well-established approach in targeted therapy. The MEK-inhibitor (MEKi) Trametinib, a non-ATP-competitive inhibitor of the MAP2K1 and MAP2K2 kinases, has demonstrated efficacy and activity in this context^45,46^. We explored additional dependencies of *NRAS*-mRNA expression and MEK signaling by treating D04 and MM415 cells with increasing Trametinib concentrations, followed by analysis of changes in *NRAS*-mRNA expression. The cells responded with a strong *NRAS*-mRNA upregulation (Fig. 4c, up to 10-fold increase in D04 and 3-fold increase in MM415 cells). This co-regulatory response may also exist in cells that are chronically exposed to Trametinib, as *NRAS*-mRNA expression was upregulated 2-fold when comparing the Trametinib-resistant *NRAS*-mutant melanoma cell lines D04RM and MM415RM to their according parental cell lines from which they had originated (Fig. 4d, resistance was acquired through chronic exposure to increasing Trametinib concentrations. The cell lines are referred to using the “RM” suffix; see methods for details). However, acquired Trametinib resistance did not alter the cells’ vulnerability to NRAS ASO-1 treatment, as the growth inhibition of 4 resistant cell lines was comparable to their Trametinib sensitive parental cell lines (Figs. 3a,4e). Building on these regulatory dependencies, we explored potential favorable therapeutic implications of the combination of NRAS ASO-1 and Trametinib treatment. We administered NRAS ASO-1 combined with a wide range of Trametinib concentrations to D04 and MM415 cells. The antiproliferative effects of the dual treatment regimen were analyzed by calculating the Highest single agent (HSA) synergy scores (see method section for detailed information). Notably, the combined treatment of NRAS ASO-1 and Trametinib consistently exhibited synergistic effects, measured for treatment periods of three and five days (Fig. 4f−i).

**Fig. 5:**
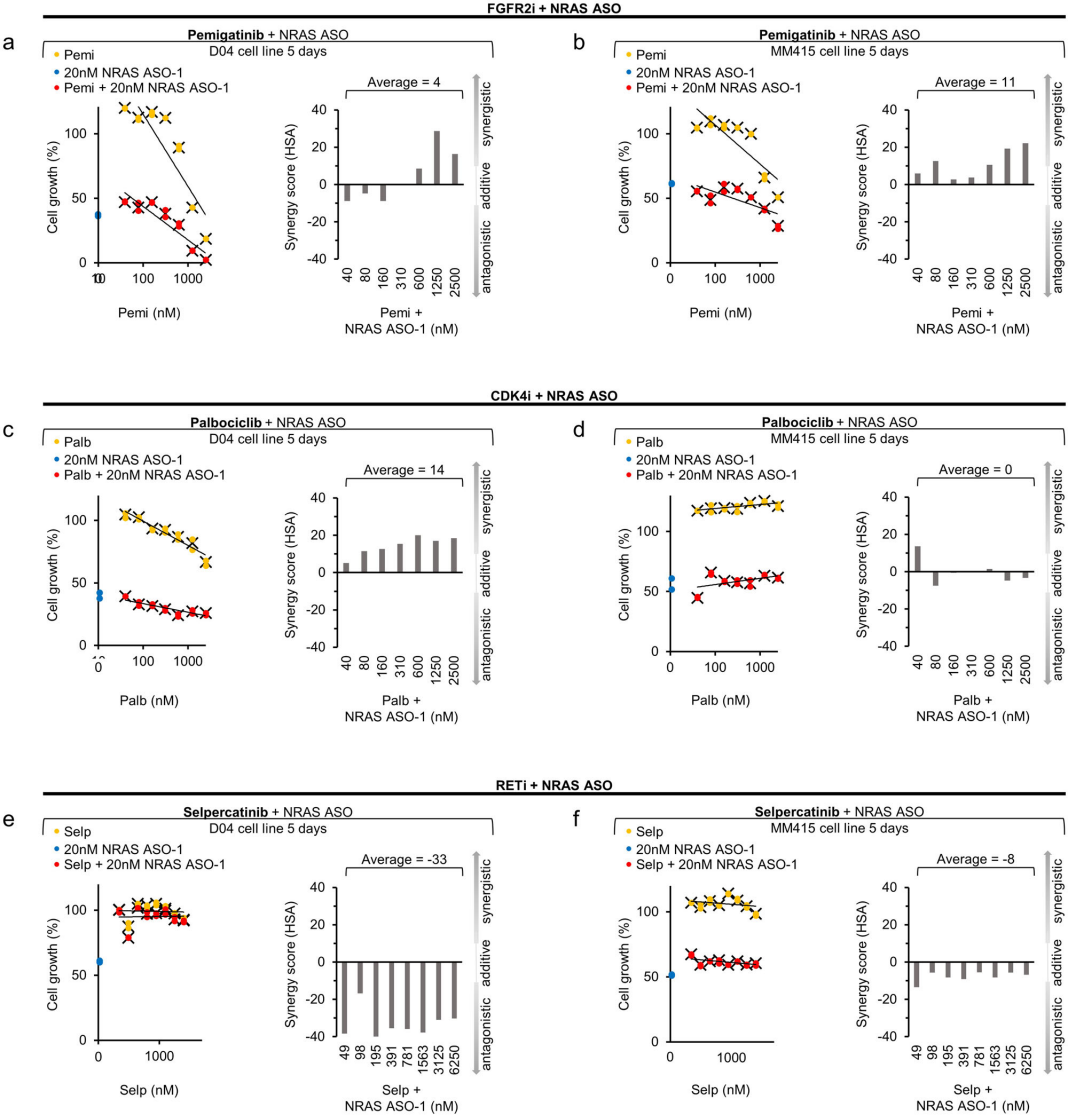
NRAS ASO-1 treatment in combination with FGFR2 and CDK4 inhibition has synergistic potential. **a**–**d** Dual treatment with 20 nM of NRAS ASO-1 and the FGFR2 inhibitor pemigatinib (Pemi, **a**, **b**, 40 nM –2500 nM), respectively the CDK4 inhibitor palbociclib (Palb, **c**, **d**, 40 nM–2500 nM) caused synergistic effects in the D04 (**a** + **c**) and MM415 (**b**) cell lines after 5 days of treatment. Dual treatment with NRAS ASO-1 and Palbociclib caused additive overall effects in MM415 (**d**), with synergism in the lowest Palbociclib dose regimen (40 nM). Dual treatment with 20 nM of NRAS ASO-1 and the RET inhibitor selpercatinib (Selp, **e**, **f**, 49 nM - 6250 nM), caused antagonistic effects in the D04 (**e**) and MM415 (**f**) cell lines after 5 days of treatment. Dose response curves show NRAS ASO-1 treatment (blue), pemigatinib or palbociclib treatment (yellow) and dual treatment (red). Synergism of dual cell growth inhibition is shown as bar graphs and determined by the HSA synergy score (n = 2).

Next, we tested additional drugs that target kinases identified by HT-KAM, and that may also synergize with NRAS ASO-1 treatment. Combinatorial kinase targeting therapies, including inhibitors of FGFR and CDK4/6, have been studied to treat melanoma^3,47,48^. We evaluated the potential synergistic effects of NRAS ASO-1 in combination with FGFR2-inhibition using the small molecule inhibitor pemigatinib, which is an FDA approved inhibitor to treat locally advanced-, FGFR2-mutated cholangiocarcinoma^49^. The combination of NRAS ASO-1 and pemigatinib also induced synergistic antiproliferative response in D04 and MM415 cells (Fig. 5a, b). A similar synergistic antiproliferative response for FGFR2-inhibition and NRAS ASO-1 treatment was observed in the MM415RM MEKi-resistant cells, regardless of whether these cells maintained in their standard MEKi dose (55 nm), or in MEKi-free media (Supplementary Fig. 2c, d). Based on the NRAS ASO-1 induced change in kinase activity profile, we also tested the combination of CDK4-inhibiton and NRAS ASO-1. For this purpose, we used palbociclib, which is an FDA approved CDK4/6 inhibitor for the treatment of ER + , and HER2- Metastatic Breast Cancer^50^. This combination caused synergetic effects in the D04 cell line (Fig. 5c). However, singular treatment with palbociclib did not reduce proliferation in MM415 cells. Instead, it even increased the proliferation rate of MM415 cells in the range of tested concentrations. In combination with NRAS ASO-1, the proliferation inducing effect of palbociclib was inhibited and synergistic and additive effects were observed in the dual regimen (Fig. 5d). The kinase inhibitors promoted cell growth when used alone at very low concentrations (Fig. 4f−i,5a−d). This was particularly evident with palbociclib, which increased cell growth in the MM415 cell line at all tested concentrations. However, this proliferative effect was reduced or even neutralized by the additional application of NRAS-ASO-1.

RET kinase activity was significantly downregulated following NRAS ASO treatment (Fig. 4b, *p* = 0.012). To further assess the specificity of HT-KAM in identifying upregulated kinase activity with synergistic dual treatment potential, we tested whether the RET inhibitor, Selpercatinib, may inhibit the effects of NRAS-ASO treatment. The combination of NRAS ASO-1 and selpercatinib induced antagonistic responses in D04 and MM415 cells, with an almost complete inhibition of the NRAS ASO-1 effect in D04 (Fig. 5e, f).

In summary, the HT-KAM analysis of kinase activity shifts induced by NRAS ASO-1 treatment enabled the identification of specific kinases potentially involved in the rescue mechanisms triggered by NRAS inhibition. Co-targeting NRAS and the identified kinases resulted in a synergistic antiproliferative effect in five out of six instances and an additive effect in one out of six instances.

## Discussion

In the history of melanoma treatment, substantial strides have been achieved with targeted therapy, particularly benefiting the largest group of patients with *BRAF* mutations^3^. In contrast, *NRAS*-mutant melanoma poses unique therapeutic challenges and is characterized by resistance to existing targeted therapies and aggressive tumor growth^3^. In the absence of a direct NRAS inhibitor of the “small molecule” type^7^, we tested chemically modified GapmeR ASOs to directly and specifically target and deplete *NRAS*-mRNA. NRAS ASOs induced apoptosis and efficiently suppressed *NRAS* expression and MAPK-pathway signaling. Most notably, the ASOs inhibited *NRAS*-mutant melanoma growth in vitro and in vivo. Our findings underscore that the maintenance of unaltered *NRAS*-mRNA expression may be a specific vulnerability of *NRAS*-mutant melanoma cells, eliminating the need to solely focus on the inhibition of mutant NRAS. Additionally, the analysis of the changes in kinase activity upon NRAS ASO treatment allowed us to identify a specific set of kinase inhibitors that enhanced the therapeutic impact of NRAS ASOs in dual treatment strategies.

The most widely employed methods for targeting and depleting RNA in research and clinical applications are ASOs and siRNAs^11,34^. ASO-mediated degradation of *NRAS*-mRNA partially outperformed siRNA-mediated degradation in the context of this study. We present factors indicating that ASO-mediated depletion of *NRAS*-mRNA may bare certain advantages compared to siRNA. Unlike siRNAs, ASOs can exhibit their activity independent of the subcellular localization of their target^14,35^. This advantage may address the challenge of targeting *NRAS*-mRNA, which we found to be present in both nuclear and cytoplasmic compartments of *NRAS*-mutant melanoma cells. SiRNAs are more likely to induce immune related toxicity^51^, and their design necessitates the additional consideration of potential side effects arising from an active guide strand^34^. Additionally, ASOs allow certain flexibilities in target sequence design, because they can target intronic mRNA regions and do not require complementary strands for cellular delivery^14,34^. Our research extends previous efforts that used siRNAs to mainly explore the role of NRAS in melanoma related signal transduction and cell cycle regulation^21,22,52^. Previous studies involving NRAS-mutant melanoma focused on targeting the mutational sites of point-mutated oncogenic *NRAS*-mRNA^21,22^. Our studies suggest a broader approach. Recognizing the independence of most non-cancerous cells on *NRAS* expression, we highlight the advantage of using the entire *NRAS*-mRNA sequence for ASO design. This approach allows testing of a greatly increased array of oligonucleotide sequences that target accessible mRNA regions. We designed two *NRAS*-targeting ASOs and evaluated that both meet the requirements of specific and efficient targeting of *NRAS*-mRNA, with NRAS ASO-1 causing stronger reductions of *NRAS*-mRNA levels than NRAS ASO-2. Both ASOs induced highly similar effects in regards of cell growth inhibition, outperforming ASOs that target the mutational mRNA region in NRAS^Q61L^-mutated melanoma cells.

The observation that the absolute extent of *NRAS*-mRNA expression may not necessarily stand in a strong correlation with antiproliferative responses to NRAS ASO treatment, further expanded to cell lines that acquired resistance to the MEK inhibitor (MEKi) Trametinib. Trametinib resistance triggered increased *NRAS*-mRNA expression in these cell lines, but the inhibiting effects of NRAS ASO treatment (50 nM ASO concentration) were highly similar when compared to their naïve parental cell lines.

Additionally, we identified critical regulatory relationships between *NRAS*-mRNA expression and the activity of several kinases. Kinome activity profiling of NRAS ASO-1 treated melanoma cells revealed MEK1 as the most significantly upregulated kinase in response to NRAS ASO treatment, which was therapeutically exploitable. Dual treatment with NRAS ASO-1 and Trametinib (MEKi) was synergistic and amplified the antiproliferative effects. When MEKi was previously compared to the long-standing but limitedly proven cytostatic dacarbazine, only modest responses were observed in the treatment of *NRAS*-mutant melanoma^9,53^, and a clinical trial indicated that *NRAS*-mutant melanoma patients only show limited benefit to MEKi^54^. In accordance with the observed synergy with NRAS ASOs, promising results were obtained when MEKi was combined, with other MAPK-signaling inhibitors^55^, including dual treatment with KRAS inhibitors as currently tested in a clinical trial for *KRAS*-mutated advanced solid tumors (ClinicalTrials.gov ID NCT04185883). Expanding the dual treatment testing to the other two most upregulated kinases, FGFR2 and CDK4 also resulted in synergistic and additive antiproliferative effects. The observed synergism further aligns with prior reports of promising pre-clinical and clinical data of dual MEK and CDK4/6 targeting for the treatment of *NRAS*-mutant melanoma^47,56^, and dual FGFR- and BRAF-inhibition for the treatment of *BRAF*-mutant melanoma^48^.

In some cases, we observed that low concentrations of the kinase inhibitors promoted melanoma cell growth. Similar patterns in dose-response curves have been observed for low-dose MEK, FGFR, and CDK inhibitors^57–59^ –, although to our knowledge, this phenomenon has not been directly addressed in the literature. Drug induced pathway inhibition can cause compensatory upregulation of associated pro-survival pathways^60^. One plausible explanation for the observed effects may be that at suboptimal drug concentrations the targets are not fully inhibited, but bypass mechanisms that promote growth are already activated. NRAS-ASO-1 treatment either diminished or even reversed this stimulation of cell growth, further underscoring a potential therapeutic benefit of NRAS ASOs in dual treatment regimen.

Given the unsatisfactory results of small molecule inhibitor treatment in the specific focus of *NRAS*-mutant melanoma, our findings suggest that improvement may involve combinatorial approaches that include the inhibition of NRAS.

It has been shown that NRAS signaling blocks apoptosis in melanoma^52,61^. NRAS had been investigated as a target for melanoma therapy, but no NRAS-targeting small molecule inhibitor has been approved for clinical approaches^3,7^. Indirect NRAS inhibition by farnesyl transferase inhibitors reduced the growth of melanoma, but caused strong toxicity due to the unspecific inhibition of other proteins^42^. Direct targeting of mRNA of other RAS gene family members showed strong potential in regards of antitumor activity, leading to clinical trials of an mRNA-based cancer vaccine, an ASO, and two different siRNAs for targeting KRAS in cancer patients (ClinicalTrials.gov IDs: NCT03948763, NCT03101839, NCT01188785, NCT01676259 and NCT03608631).

Accounting for off-target related hepatotoxicity that can occur with certain ASOs of the GapmeR type^62^, we validated that the NRAS ASO treatment did not significantly alter liver function parameters in mice, and was not toxic to primary derived human liver cells. Athymic nude mice, widely recognized as standard model for immunodeficient hosts for tumor transplantation and drug efficacy testing in oncology, were utilized for in vivo experiments^63^. Subcutaneous injections were utilized as the route of administration, a validated method for delivering ASOs systemically, with pharmacokinetic profiles comparable to intravenous administration^64^.

The RAS gene family, consisting of *NRAS*, *KRAS*, and *HRAS* encodes small GTPases that function as molecular switches in regulating critical cell signaling pathways involved in proliferation, differentiation, and survival^65^. Despite their high sequence similarity, they display distinct biological roles and tissue-specific expression patterns^65^. The interplay between NRAS, KRAS and HRAS in the context of *NRAS*-mutant melanoma and other tumor types remains poorly understood. Our findings demonstrate that treatment with NRAS-targeting ASOs had minimal impact on the mRNA expression of *KRAS* or *HRAS*, which highlights the specific effects of the treatment.

Consistent cross-comparison to treatment with non-targeting control ASOs, the analysis of publicly available *NRAS*-knockout databases and the thorough in vitro and in vivo testing on cancerous and non-cancerous cells, further suggested a highly specific and targeted impact of NRAS ASOs on *NRAS*-mutant melanoma cells and low toxicity on *NRAS*-WT cells. Further evaluation of toxic side effects of systemic NRAS ASO treatment in mice was limited by the circumstance that the NRAS ASO sequence specifically targets human NRAS. The translation of ASO treatment into clinical settings presents other challenges besides toxic side effects, such as limitations of systemic delivery^51^. Our approach to mitigate these challenges involved using GapmeR ASOs, which incorporate LNAs and utilize a fully modified phosphorothioate (PS) backbone. These modifications greatly enhance binding affinity, stability, and target specificity. While there are ways to further improve ASO delivery with additional chemical modifications, or nanoparticle-mediated delivery, these aspects remain beyond the scope of the current study. Cancer driving *NRAS* mutations extend beyond melanoma. Our study hints at the sensitivity of other *NRAS*-mutant cancer cells to NRAS ASO treatment, which may be addressed independently.

Next-generation-sequencing techniques (NGS) allow to identify tumor driving mutations in short time and at low costs^66^, and the detection of cancer-driving mutations and their accompanying vulnerabilities to certain treatment regimen is a main approach in modern medicine^67^. Our findings indicate that the mutation status of *NRAS* could serve as a prognostic biomarker for the efficiency of NRAS targeted therapy, which bears the potential for additional amplification by combinatorial treatment with specific kinase inhibitors.

## Supplementary information

Supplementary Information

Description of Additional Supplementary Files

Supplementary Data 1

Supplementary Data 2

REPORTING SUMMARY

## Data Availability

The dependency datasets analyzed in this study (Fig. 1a, b) are obtained from the Dependency Map Portal (https://depmap.org/portal/), and provided in Supplementary Data 1. The source data for Figs. 1c–f, 2a, 3a–c, 3i–d, g–h, j–l, 4b–i, and 5a–f are provided in Supplementary Data 2. Uncropped western blot images are provided in Supplementary Fig. 3. The gating strategy for flow cytometry (Fig. 3f) is provided in Supplementary Fig. 4. Additional data supporting the findings of this study are available on request from the corresponding author, V.F.
