# Supplementary material for: Suppression of NRAS-mutant melanoma growth with NRAS-targeting Antisense Oligonucleotide treatment reveals therapeutically relevant kinase co-dependencies
Source: Commun Med (Lond). 2025 Jun 5;5:216. doi: 10.1038/s43856-025-00932-5 (PMC12141655; doi:10.1038/s43856-025-00932-5)
Supplement: Supplementary file 1 — Supplementary Information [file 43856_2025_932_MOESM1_ESM.pdf]

Suppression of *NRAS*-mutant melanoma growth with NRAS-targeting Antisense Oligonucleotide treatment reveals therapeutically relevant kinase co-dependencies

Valentin Feichtenschlager[1,2*], Yixuan James Zheng[1,3], Tiange Qu[4], Dasha Hohlova[1,5], Ciara Callanan[1], Linan Chen[1], Christopher Chen[1], Wilson Ho[1], Albert Lee[1], Yeonjoo Hwang[6], Arowyn Courtright[1], Thy Nguyen[1], Olivia Marsicovetere[1], Denise P. Muñoz[6], Klemens Rappersberger[2], Jean-Philippe Coppe[7], Susana Ortiz-Urda[1]

Affiliations:

[1] Department of Dermatology, Mt Zion Cancer Research Center, University of California San Francisco, San Francisco, CA, USA

[2] Department of Dermatology, Clinic Landstrasse Vienna, Academic Teaching Hospital, Medical University Vienna, Vienna, Austria

[3] School of Medicine, University of California San Francisco, San Francisco, CA, USA

[4] Department of Orofacial Science, Health Science West, University of California San Francisco School of Dentistry, San Francisco, CA, USA

[5] Department of Biology, University of San Francisco, San Francisco, CA, USA

[6] Department of Hematology-Oncology, Helen Diller Family Comprehensive Cancer Center, University of California San Francisco, San Francisco, CA, USA

[7] Department of Radiation Oncology, Helen Diller Family Comprehensive Cancer Center, University of California San Francisco, San Francisco, CA, USA

*Corresponding Author.

Email: valentin.feichtenschlager@hotmail.com

Supplementary Information

Supplementary Figures, Supplementary Tables

Supplementary Figures

Supplementary Figure 1

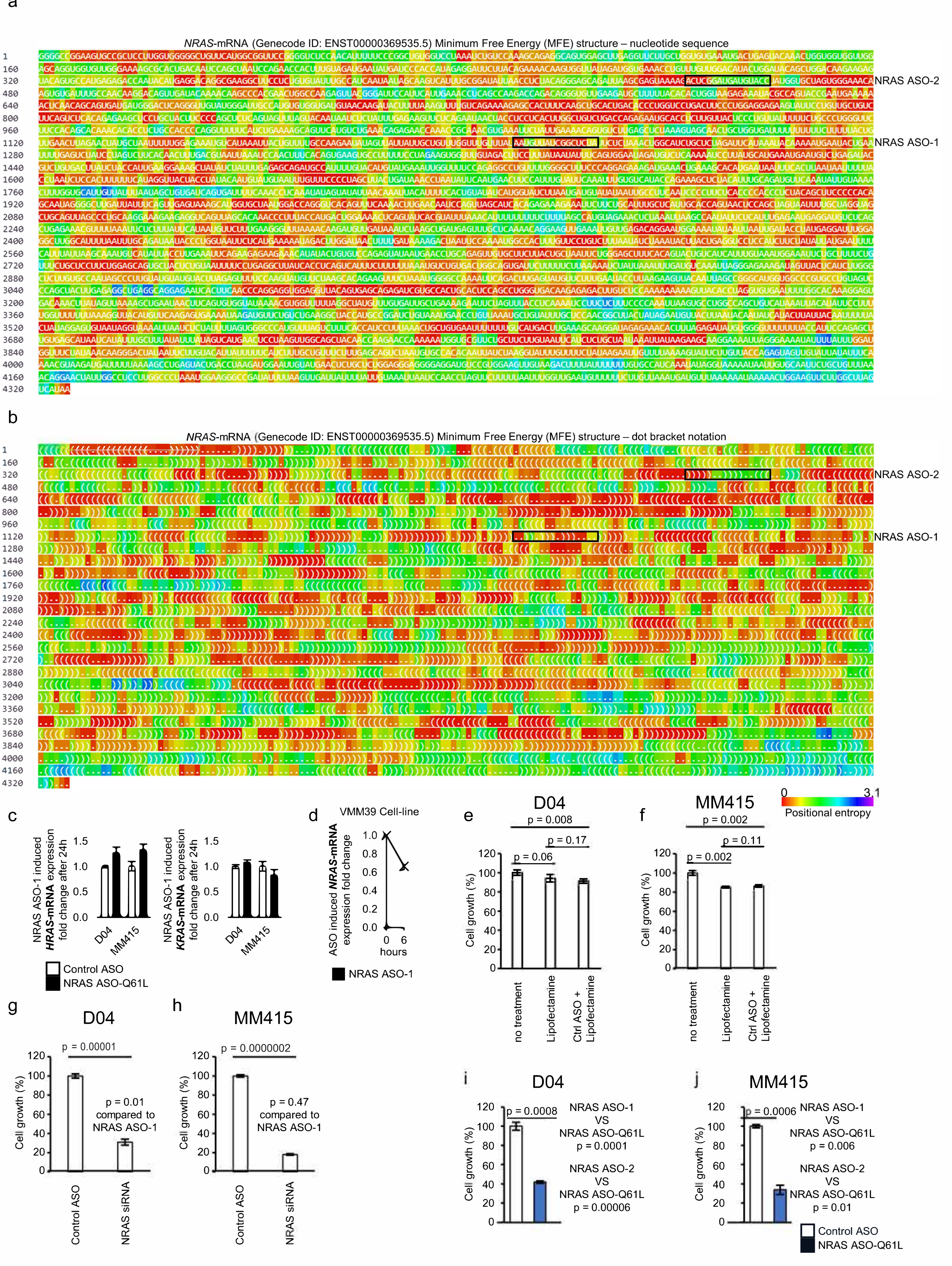

Supplementary Figure 1. a) Linear representation of the optimal secondary structure of the NRAS-mRNA (Genecode ID: ENST00000369535.5) nucleotide sequence, color coded for positional entropy. The minimum free energy (MFE) is -1130.47 kcal/mol. b) Linear representation of the optimal secondary structure of the NRAS-mRNA in dot-bracket notation, color coded for positional entropy. The characters “(“ and “)” correspond to the 3’ base and 5’ base in the base-pair, while “.” corresponds to an unpaired base. The NRAS ASO target regions are annotated with a black frame. c) Using qRT-PCR to compare RNA levels in D04 and MM415 cells that were either treated with NRAS ASO-1 or Control ASO, showed a limited increase of HRAS-mRNA levels, and minimal to no impact on KRAS-mRNA levels after 24 hours of treatment. Final oligonucleotide concentration was 100 nM; error bars represent s.e.m. (n=3). d) Using qRT-PCR to compare RNA levels in VMM39 cells that were treated with NRAS ASO-1, shows a reduction of NRAS-mRNA levels after 6 hours, when compared to treatment with non-targeting Control ASO. Final oligonucleotide concentration was 100 nM. e-f) Treatment with Control ASO and the transfection reagent Lipofectamine 3000 caused a significant but limited inhibition of cell growth in the NRAS-mutant melanoma cell lines D04 (-9%) and MM415 (-14%), when compared to untreated cells. Treatment with Lipofectamine alone (excluding ASO) also reduced cell growth in both cell lines (D04: -6%; MM415: -15%). g-h) Treatment with NRAS siRNA caused significant inhibition of cell growth in the D04 (-69%) and MM415 (-82%) cell lines. i-j) Treatment with NRAS ASO-Q61L caused significant inhibition of cell growth in the D04 (-58%) and MM415 (-66%) cell lines. For e-j) the final oligonucleotide concentration was 50 nM, treatment period was 5 days (n=3). The error bars in d) represent s.e.m, in e-j) they represent s.d. Significance is shown as p-values calculated by Student’s t-test. * = p < 0.05, ** = p < 0.01, *** = p < 0.001.

Supplementary Figure 2

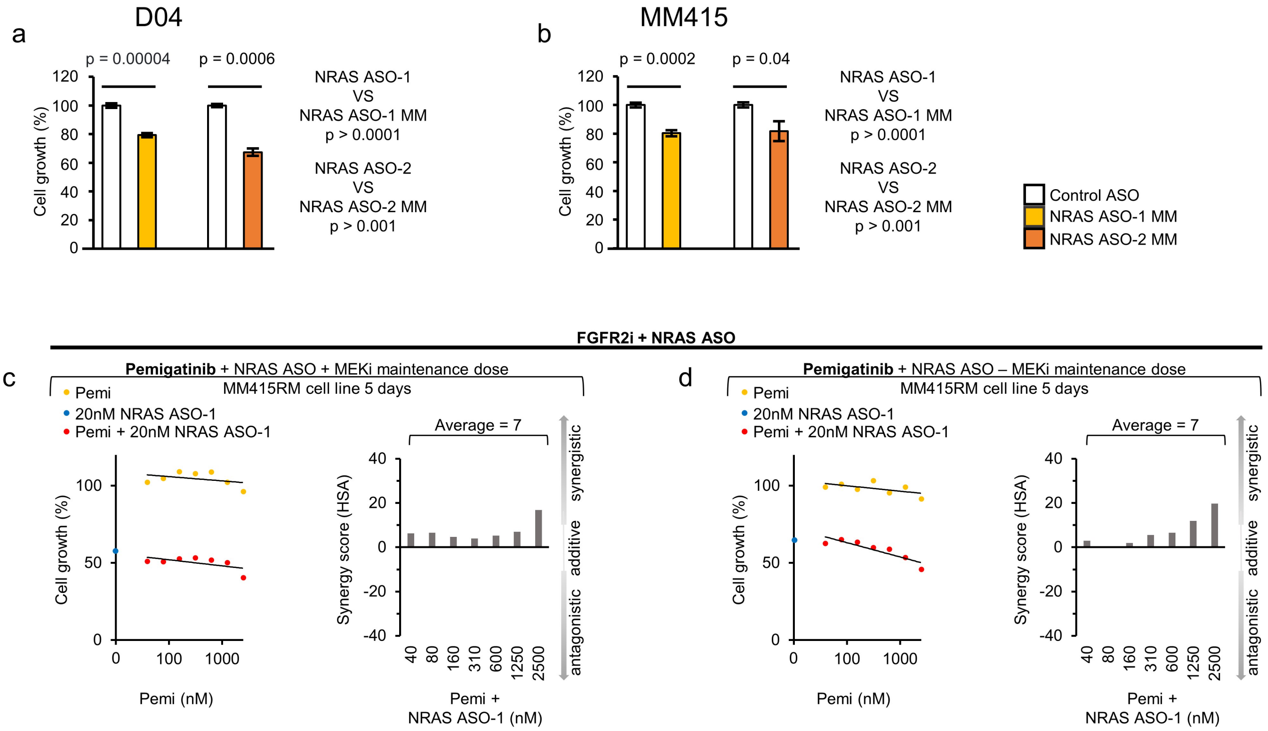

Supplementary Figure 2. a-b) Treatment with modified NRAS ASO 1+2, harboring one mismatch to the NRAS-mRNA sequence (NRAS ASO-1+2 MM) caused significant less inhibition of cell growth in the D04 and MM415 cell lines, when compared to their original ASO versions (n=3). c-d) Dual treatment with 20nM NRAS ASO-1 and the FGFR2 inhibitor pemigatinib (Pemi, 40 nM -2500 nM) resulted in synergistic effects in the MEKi-resistant MM415RM cell line after 5 days of treatment (n=2). This synergy was observed regardless of whether the treatment media included the cells’ MEKi-maintenance dose (c), or was MEKi free (d).

Supplementary Figure 3

a

Fig. 2d D04

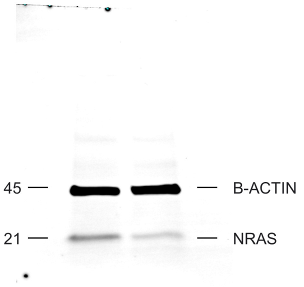

b

Fig. 2d MM415

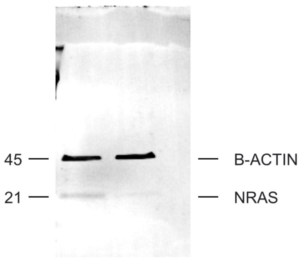

c

Fig. 2e D04+MM415

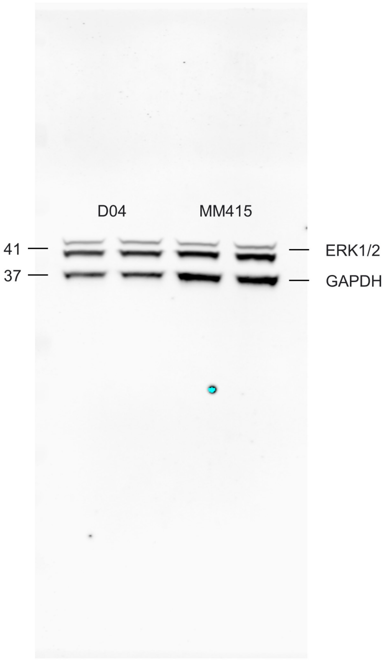

d

Fig. 2e D04+MM415

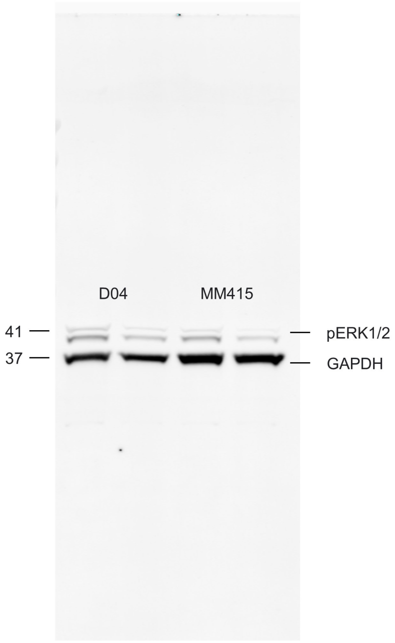

e

Fig. 2g D04

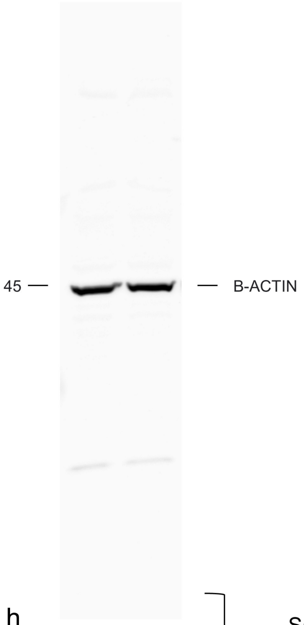

f

Fig. 2g MM415

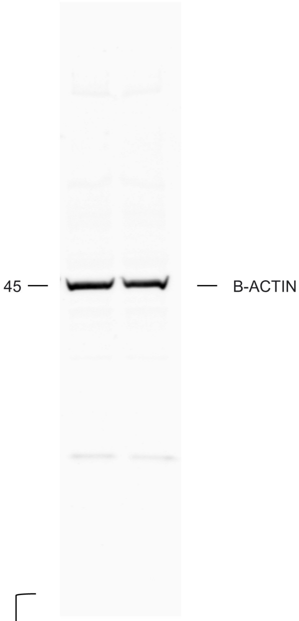

g

Fig. 2g D04+MM415

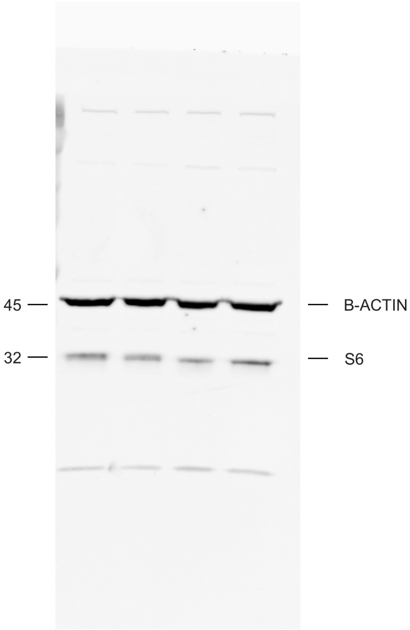

h

Fig. 2g D04

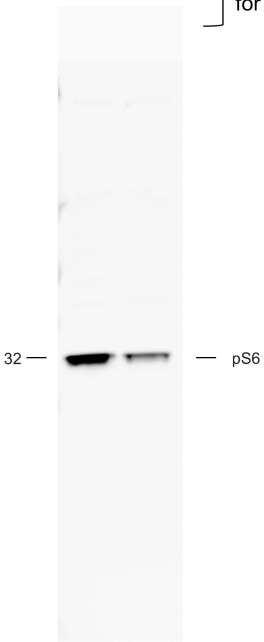

Same membrane, individual scans for each channel

i

Fig. 2g MM415

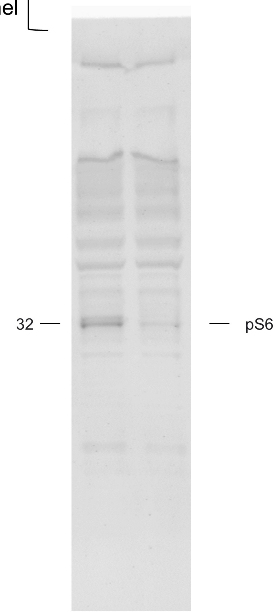

j

Fig. 2f D04+MM415

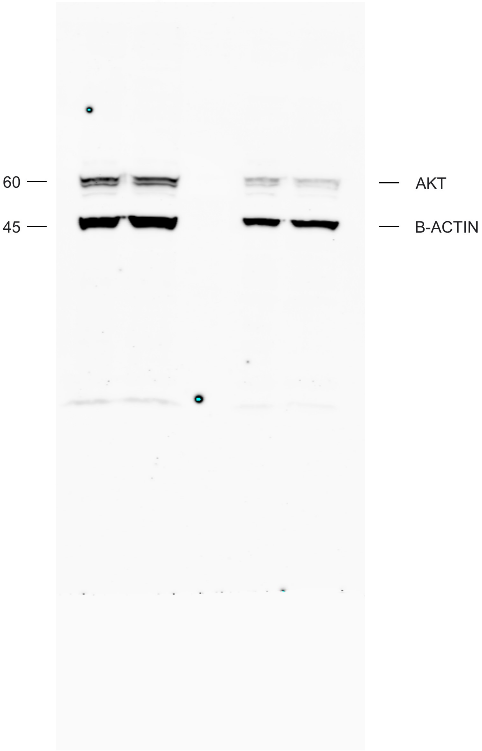

k Fig. 2f D04+MM415

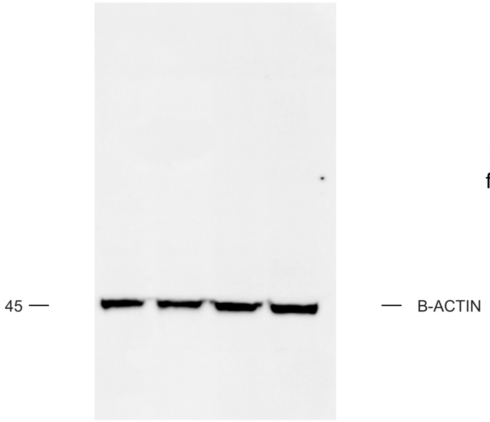

Same membrane, individual scans for each channel

l Fig. 2f D04+MM415

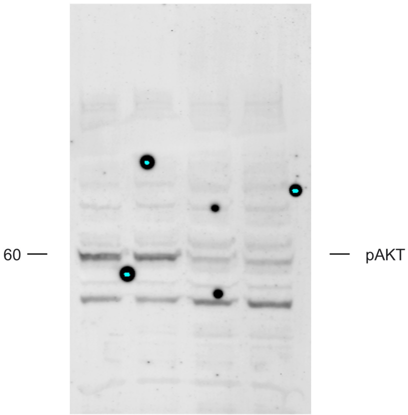

Supplementary Figure 3. a-b) Uncropped western blot images for figure 2d. c-d) Uncropped western blot images for figure 2e. e-i) Uncropped western blot images for figure 2g. j-l) Uncropped western blot images for figure 2f.

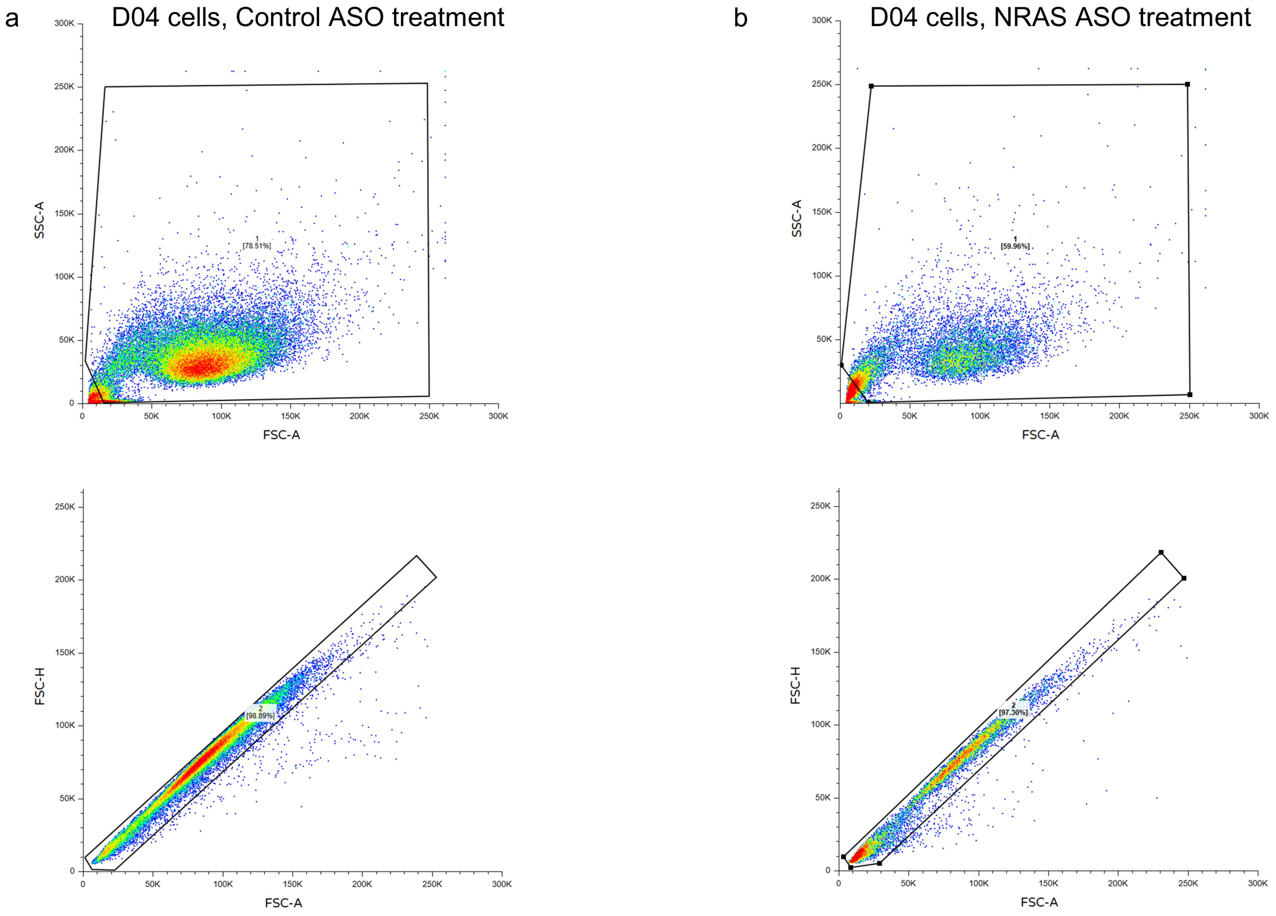

Supplementary Figure 4. a-b) Gating strategy for flow cytometry. Gating was performed to ensure the correct cell populations were used for analysis. As the first step, cells were gated based on side scatter area (SSC-A) vs forward scatter area (FSC-A). To exclude cellular debris, cells with low SSC-A and FSC-A signals excluded. Next, cells were gated based on forward scatter height (FSC-H) vs FSC-A to exclude doublets or aggregates from the data. There appeared to be minimal doublets in the cell populations. Finally, as shown in Fig. 3f, the remaining cells were gated based on Annexin V and PI. Low Annexin V and low PI represent viable cells (Q2), high Annexin V and low PI represent apoptotic cells (Q3), and high Annexin V and high PI represent late apoptotic and necrotic cells (Q4).

Supplementary Tables

Construct name	Target sequence and chemical modifications
NRAS ASO-1	5’- +T*+A*+G*A*G*C*C*G*A*T*A*A*C*+A*+T*+T -3’
NRAS ASO-2	5’- +G*+G*+T*A*C*A*T*C*A*T*C*C*G*+A*+G*+T -3’
NRAS ASO-1 MM	5’- +T*+A*+G*A*G*C*C*G*C*T*A*A*C*+A*+T*+T -3’
NRAS ASO-2 MM	5’- +G*+G*+T*A*C*C*T*C*A*T*C*C*G*+A*+G*+T -3’
NRAS ASO-Q61L	5'- +G*+T*+A*C*T*C*T*T*C*T*A*G*T*+C*+C*+A -3'
NRAS siRNA (pool)	GAGCAGAUUAAGCGAGUAA, GAAAUACGCCAGUACCGAA, GUGGUGAUGUAACAAGAUA, GCACUGACAAUCCAGCUAA

Supplementary Table 1. Sequence and structure of NRAS-targeting constructs. GapmeR constructs contain phosphorothioate backbone modifications indicated by “*”, and LNA modifications indicated by “+”. Mismatch exchange nucleotides are highlighted in red.

Rank	Transcript name	species	Accession Nr	Total Score	E-value	miss matches
1	NRAS	Homo Sapiens	NM_002524.5	32.2	1.5	0
2	EMC10-2	Homo Sapiens	NM_206538.4	26.3	90	3
3	B3GALT5-6	Homo Sapiens	NM_001278650.2	26.3	90	3
4	B3GALT5-4	Homo Sapiens	NM_033172.3	26.3	90	3
5	B3GALT5-1	Homo Sapiens	NM_006057.3	26.3	90	3
6	B3GALT5-9	Homo Sapiens	NM_001356339.2	26.3	90	3
7	EMC10-1	Homo Sapiens	NM_175063.6	26.3	90	3
8	B3GALT5-7	Homo Sapiens	NM_001356336.2	26.3	90	3
9	B3GALT5-8	Homo Sapiens	NM_001356338.2	26.3	90	3
10	B3GALT5-2	Homo Sapiens	NM_033170.3	26.3	90	3
11	B3GALT5-3	Homo Sapiens	NM_033171.3	26.3	90	3
12	STAU2-7	Homo Sapiens	NM_001164385.2	24.3	354	4
13	STAU2-5	Homo Sapiens	NM_014393.3	24.3	354	4
14	CEP44-2	Homo Sapiens	NM_001145314.2	24.3	354	4
15	STAU2-6	Homo Sapiens	NM_001164384.2	24.3	354	4
16	COX11-1	Homo Sapiens	NM_004375.5	24.3	354	4
17	COX11-5	Homo Sapiens	NR_027942.3	24.3	354	4
18	COX11-4	Homo Sapiens	NR_027941.3	24.3	354	4
19	COX11-7	Homo Sapiens	NR_135677.2	24.3	354	4
20	WDR3	Homo Sapiens	NM_006784.3	24.3	354	4

Supplementary Table 2. Comparison of the NRAS ASO-1 sequence TAGAGCCGATAACATT to its top 20 hits (ranked by e-value) in the human transcriptome shows high on-target affinity to the NRAS RNA and low off-target binding affinity to other RNA-transcripts.

Rank	Transcript name	species	Accession Nr	Total Score	E-value	miss matches
1	NRAS	Homo Sapiens	NM_002524.5	32.2	1.5	0
2	TCEAL4-6	Homo Sapiens	NM_001305840.2	24.3	354	4
3	TCEAL4-5	Homo Sapiens	NM_001300901.2	24.3	354	4
4	DOC2A-2	Homo Sapiens	NM_001282063.2	24.3	354	4
5	MLEC-2	Homo Sapiens	NM_001303627.2	24.3	354	4
6	TFIP11-5	Homo Sapiens	NM_001346859.2	24.3	354	4
7	TFIP11-3	Homo Sapiens	NM_001346857.2	24.3	354	4
8	TCEAL4-8	Homo Sapiens	NM_001305842.2	24.3	354	4
9	TCEAL4-7	Homo Sapiens	NM_001305841.2	24.3	354	4
10	TFIP11-6	Homo Sapiens	NM_001346861.2	24.3	354	4
11	TCEAL4-4	Homo Sapiens	NM_001006937.3	24.3	354	4
12	TFIP11-1	Homo Sapiens	NM_001008697.3	24.3	354	4
13	TCEAL4-1	Homo Sapiens	NM_024863.6	24.3	354	4
14	TFIP11-4	Homo Sapiens	NM_001346858.2	24.3	354	4
15	TCEAL4-2	Homo Sapiens	NM_001006935.3	24.3	354	4
16	SHANK3	Homo Sapiens	NM_001372044.2	24.3	354	1
17	DMXL2-13	Homo Sapiens	NR_165649.1	24.3	354	4
18	DMXL2-12	Homo Sapiens	NR_165648.1	24.3	354	4
19	DMXL2-2	Homo Sapiens	NM_015263.5	24.3	354	4
20	DMXL2-1	Homo Sapiens	NM_001174116.3	24.3	354	4

Supplementary Table 3. Comparison of the NRAS ASO-2 sequence GGTACATCATCCGAGT to its top 20 hits (ranked by e-value) in the human transcriptome shows high on-target affinity to the NRAS RNA and low off-target binding affinity to other RNA-transcripts.

Rank	Transcript name	species	Accession Nr	Total Score	E-value	miss matches
1	COL25A1	Homo Sapiens	NR_160939.1	26.3	100	2
2	LINC00645	Homo Sapiens	NR_039992.2	26.3	100	2

Supplementary Table 4. Comparison of the non-targeting Control ASO sequence AACACGTCTATACGC to its top 2 hits (ranked by e-value) in the human transcriptome shows low off-target binding affinity to RNA-transcripts.

Rank	Transcript name	species	Accession Nr	Total Score	E-value	miss matches
1	PDE4DIP-49	Homo Sapiens	NM_001395426.1	26.3	90	3
2	PDE4DIP-30	Homo Sapiens	NM_001395311.1	26.3	90	3
3	PDE4DIP-33	Homo Sapiens	NM_001395314.1	26.3	90	3
4	PDE4DIP-9	Homo Sapiens	NM_001198834.5	26.3	90	3
5	PDE4DIP-14	Homo Sapiens	NM_001377392.2	26.3	90	3
6	PDE4DIP-22	Homo Sapiens	NM_001395303.1	26.3	90	3
7	PDE4DIP-11	Homo Sapiens	NM_001350521.4	26.3	90	3
8	PDE4DIP-20	Homo Sapiens	NM_001395301.1	26.3	90	3
9	PDE4DIP-13	Homo Sapiens	NM_001350523.3	26.3	90	3
10	PDE4DIP-23	Homo Sapiens	NM_001395304.1	26.3	90	3
11	PDE4DIP-17	Homo Sapiens	NM_001395298.1	26.3	90	3
12	PDE4DIP-12	Homo Sapiens	NM_001350522.3	26.3	90	3
13	PDE4DIP-8	Homo Sapiens	NM_001198832.4	26.3	90	3
14	PDE4DIP-26	Homo Sapiens	NM_001395307.1	26.3	90	3
15	PDE4DIP-16	Homo Sapiens	NM_001395297.1	26.3	90	3
16	PDE4DIP-31	Homo Sapiens	NM_001395312.1	26.3	90	3
17	PDE4DIP-24	Homo Sapiens	NM_001395305.1	26.3	90	3
18	PDE4DIP-21	Homo Sapiens	NM_001395302.1	26.3	90	3
19	PDE4DIP-18	Homo Sapiens	NM_001395299.1	26.3	90	3
20	PDE4DIP-29	Homo Sapiens	NM_001395310.1	26.3	90	3
	NRAS	Homo Sapiens	NM_002524.5	24.3	355	1

Supplementary Table 5. Comparison of the NRAS ASO-Q61L sequence GTACTCTTCTAGTCCA to its top 20 hits (ranked by e-value) in the human transcriptome and NRAS mRNA shows low off-target binding affinity to other RNA-transcripts.

Rank	Transcript name	species	Accession Nr	Total Score	E-value	miss matches
1	BICRAL-3	Homo Sapiens	NM_001393499.1	24.3	355	4
2	BICRAL-2	Homo Sapiens	NM_015349.3	24.3	355	4
3	BICRAL-1	Homo Sapiens	NM_001318819.2	24.3	355	4
4	PPP4R3B-2	Homo Sapiens	NM_020463.4	24.3	355	4
5	ABCB7-5	Homo Sapiens	NM_001271699.3	24.3	355	4
6	ABCB7-3	Homo Sapiens	NM_001271697.3	24.3	355	4
7	ABCB7-4	Homo Sapiens	NM_001271698.3	24.3	355	4
8	PPP4R3B-3	Homo Sapiens	NM_001282850.2	24.3	355	4
9	ABCB7-2	Homo Sapiens	NM_001271696.3	24.3	355	4
10	PPP4R3B-1	Homo Sapiens	NM_001122964.3	24.3	355	4
11	NRAS	Homo Sapiens	NM_002524.5	24.3	355	1
12	ABCB7-1	Homo Sapiens	NM_004299.6	24.3	355	4

Supplementary Table 6. Comparison of the NRAS ASO-1 MM sequence TAGAGCCGCTAACATT to its top 12 hits (ranked by e-value) in the human transcriptome shows low off-target binding affinity to RNA-transcripts.

Rank	Transcript name	species	Accession Nr	Total Score	E-value	miss matches
1	NAV1-7	Homo Sapiens	NM_001389615.1	26.3	90	3
2	NAV1-1	Homo Sapiens	NM_020443.5	26.3	90	3
3	NAV1-8	Homo Sapiens	NM_001389616.1	26.3	90	3
4	NAV1-9	Homo Sapiens	NM_001389617.1	26.3	90	3
5	SERPINB8-2	Homo Sapiens	NM_198833.2	24.3	355	4
6	SERPINB8-4	Homo Sapiens	NM_001276490.2	24.3	355	4
7	CDK16-3	Homo Sapiens	NM_001170460.2	24.3	355	4
8	KIF17-1	Homo Sapiens	NM_020816.4	24.3	355	4
9	KIF17-3	Homo Sapiens	NM_001287212.2	24.3	355	4
10	KIF17-2	Homo Sapiens	NM_001122819.3	24.3	355	4
11	SERPINB8-9	Homo Sapiens	NR_145571.2	24.3	355	4
12	EHMT1-5	Homo Sapiens	NM_001354611.2	24.3	355	4
13	RPS6KA1-2	Homo Sapiens	NM_001006665.2	24.3	355	4
14	EHMT1-6	Homo Sapiens	NM_001354612.2	24.3	355	4
15	SERPINB8-3	Homo Sapiens	NM_001031848.2	24.3	355	4
16	EHMT1-4	Homo Sapiens	NM_001354263.2	24.3	355	4
17	SERPINB8-8	Homo Sapiens	NM_001348370.2	24.3	355	4
18	CDK16-2	Homo Sapiens	NM_033018.4	24.3	355	4
19	EHMT1-3	Homo Sapiens	NM_001354259.2	24.3	355	4
20	SERPINB8-6	Homo Sapiens	NM_001348368.2	24.3	355	4
	NRAS	Homo Sapiens	NM_002524.5	24.3	355	1

Supplementary Table 7. Comparison of the NRAS ASO-2 MM sequence GGTACCTCATCCGAGT to its top 20 hits (ranked by e-value) and NRAS mRNA in the human transcriptome shows low off-target binding affinity to RNA-transcripts.

Rank	Transcript name	species	Accession Nr	Total Score	E-value	miss matches
1	Celf2-49	Mus musculus	NM_001406944.1	26.3	72	3
2	Celf2-48	Mus musculus	NM_001406943.1	26.3	72	3
3	Celf2-47	Mus musculus	NM_001406942.1	26.3	72	3
4	Arhgap30	Mus musculus	NM_001005508.2	24.3	286	4
5	Or11n2	Mus musculus	NM_001011794.1	24.3	286	4

Supporting Table 8. Comparison of the NRAS ASO-1 sequence ACTCGGATGATGTACC to its top 5 hits (ranked by e-value) shows low off-target binding affinity to RNA-transcripts in the mouse transcriptome.

Primer sequences (5'-3')		
**Primer Target**	**Forward**	**Reverse**
β-actin	GGACTTCGAGCAAGAGATGG	AGCACTGTGTTGGCGTACAG
GAPDH	TGGAAGGACTCATGACCACA	GCCATCACGCCACAGTTT
NRAS	ATGACTGAGTACAAACTGGTGGT	CATGTATTGGTCTCTCATGGCAC
KRAS	GGACTGGGGAGGGCTTTCT	GCCTGTTTTGTGTCTACTGTTCT
HRAS	ATGACGGAATATAAGCTGGTGGT	GGCACGTCTCCCCATCAATG

Supplementary Table 9. Forward and reverse primer sequences used for qRT-PCR.
