# Supplementary material for: Suppression of NRAS-mutant melanoma growth with NRAS-targeting Antisense Oligonucleotide treatment reveals therapeutically relevant kinase co-dependencies
Source: Commun Med (Lond). 2025 Jun 5;5:216. doi: 10.1038/s43856-025-00932-5 (PMC12141655; doi:10.1038/s43856-025-00932-5)
Supplement: Supplementary file 2 — Description of Additional Supplementary Files [file 43856_2025_932_MOESM2_ESM.pdf]

Description of Additional Supplementary Files

File name- Supplementary Data 1

File description- NRAS-mRNA dependency datasets and additional cell line information obtained from the Dependency Map Portal (https://depmap.org/portal/).

File name- Supplementary Data 2

File description- Numerical results underlying the graphs and charts presented in the main figures
