# Supplementary material for: Suppression of NRAS-mutant melanoma growth with NRAS-targeting Antisense Oligonucleotide treatment reveals therapeutically relevant kinase co-dependencies
Source: Commun Med (Lond). 2025 Jun 5;5:216. doi: 10.1038/s43856-025-00932-5 (PMC12141655; doi:10.1038/s43856-025-00932-5)
Supplement: Supplementary file 5 — REPORTING SUMMARY [file 43856_2025_932_MOESM5_ESM.pdf]

nature portfolio

Corresponding author(s): Valentin Feichtenschlager

Last updated by author(s): 1/28/2025

Reporting Summary

Nature Portfolio wishes to improve the reproducibility of the work that we publish. This form provides structure for consistency and transparency in reporting. For further information on Nature Portfolio policies, see our Editorial Policies and the Editorial Policy Checklist.

Statistics

For all statistical analyses, confirm that the following items are present in the figure legend, table legend, main text, or Methods section.

n/a	Confirmed	
☐	☒	The exact sample size (*n*) for each experimental group/condition, given as a discrete number and unit of measurement
☒	☐	A statement on whether measurements were taken from distinct samples or whether the same sample was measured repeatedly
☐	☒	The statistical test(s) used AND whether they are one- or two-sided<br>*Only common tests should be described solely by name; describe more complex techniques in the Methods section.*
☐	☒	A description of all covariates tested
☐	☒	A description of any assumptions or corrections, such as tests of normality and adjustment for multiple comparisons
☐	☒	A full description of the statistical parameters including central tendency (e.g. means) or other basic estimates (e.g. regression coefficient) AND variation (e.g. standard deviation) or associated estimates of uncertainty (e.g. confidence intervals)
☐	☒	For null hypothesis testing, the test statistic (e.g. *F*, *t*, *r*) with confidence intervals, effect sizes, degrees of freedom and *P* value noted<br>*Give P values as exact values whenever suitable.*
☒	☐	For Bayesian analysis, information on the choice of priors and Markov chain Monte Carlo settings
☒	☐	For hierarchical and complex designs, identification of the appropriate level for tests and full reporting of outcomes
☒	☐	Estimates of effect sizes (e.g. Cohen's *d*, Pearson's *r*), indicating how they were calculated

Our web collection on statistics for biologists contains articles on many of the points above.

Software and code

Policy information about availability of computer code

Data collection	No code was used to collect data in this study
Data analysis	No code was used to analyze data in this study

For manuscripts utilizing custom algorithms or software that are central to the research but not yet described in published literature, software must be made available to editors and reviewers. We strongly encourage code deposition in a community repository (e.g. GitHub). See the Nature Portfolio guidelines for submitting code & software for further information.

Data

Policy information about availability of data

All manuscripts must include a data availability statement. This statement should provide the following information, where applicable:

- Accession codes, unique identifiers, or web links for publicly available datasets
- A description of any restrictions on data availability
- For clinical datasets or third party data, please ensure that the statement adheres to our policy

The dependency datasets analyzed in this study are obtained from the Dependency Map Portal (https://depmap.org/portal/), and provided in Supplementary Data. Additional data supporting the findings of this study are available on request from the corresponding author, V.F.

Research involving human participants, their data, or biological material

Policy information about studies with human participants or human data. See also policy information about sex, gender (identity/presentation), and sexual orientation and race, ethnicity and racism.

Reporting on sex and gender	n/a
Reporting on race, ethnicity, or other socially relevant groupings	n/a
Population characteristics	n/a
Recruitment	n/a
Ethics oversight	n/a

Note that full information on the approval of the study protocol must also be provided in the manuscript.

Field-specific reporting

Please select the one below that is the best fit for your research. If you are not sure, read the appropriate sections before making your selection.

☒ Life sciences ☐ Behavioural & social sciences ☐ Ecological, evolutionary & environmental sciences

For a reference copy of the document with all sections, see nature.com/documents/nr-reporting-summary-flat.pdf

Life sciences study design

All studies must disclose on these points even when the disclosure is negative.

Sample size	The sample size for this study was determined based on preliminary data and literature review. For in vitro experiments, 2-3 replicates were used per condition to account for biological variability and to ensure reproducibility of the results.
Data exclusions	No data were excluded
Replication	To ensure the robustness and reproducibility of our findings, all key experiments were performed in replicates. Experiments were performed independently, using separate batches of reagents and independently cultured cell lines.
Randomization	To minimize bias and ensure the validity of our findings, randomization was employed using a random number generator.
Blinding	The investigators were not blinded.

Reporting for specific materials, systems and methods

We require information from authors about some types of materials, experimental systems and methods used in many studies. Here, indicate whether each material, system or method listed is relevant to your study. If you are not sure if a list item applies to your research, read the appropriate section before selecting a response.

Materials & experimental systems

n/a	Involved in the study	
☐	☒	Antibodies
☐	☒	Eukaryotic cell lines
☒	☐	Palaeontology and archaeology
☐	☒	Animals and other organisms
☒	☐	Clinical data
☒	☐	Dual use research of concern
☒	☐	Plants

Methods

n/a	Involved in the study	
☒	☐	ChIP-seq
☐	☒	Flow cytometry
☒	☐	MRI-based neuroimaging

Antibodies

Antibodies used	NRAS (Santa Cruz Biotechnology®, cat.no.: sc-31, dilution 1:50), ERK1/2 (Cell Signaling Technology®, cat.no.:4695, dilution 1:600), p-ERK1/2 (Cell Signaling Technology®, cat.no.:4370, dilution 1:600), GAPDH (Cell Signaling Technology®, cat.no.:97166, dilution 1:1,000), p-S6 (Cell Signaling Technology®, cat.no.:4857, dilution 1:500), S6 (Cell Signaling Technology®, cat.no.:2217, dilution 1:600),

	B-ACTIN (Cell Signaling Technology®, cat.no.: 8457, dilution 1:3,000, or abcam, cat.no.:8226, dilution 1:1,000), p-Akt (Cell Signaling Technology®, cat.no.:4060, dilution 1:400), and Akt (Cell Signaling Technology®, cat.no.: 9272, dilution 1:400)
Validation	Validation statements for all antibodies can be found on the manufacturer's websites.

Eukaryotic cell lines

Policy information about cell lines and Sex and Gender in Research

Cell line source(s)	Cell lines VMM39, H929 and SW1271 were purchased from American Type Culture Collection (ATCC®). Human melanoma cell-lines D04, MM415, WM3629, Sk-Mel-2, WM3060, Sk-Mel-28 and WM1366 were gifted by Dr. Boris Bastian at the UCSF. The human melanoma cell-line NZM40 was gifted by Dr. Rony Francois at the UCSF. Primary human melanoma cell-line Hs852T, and primary human liver cells Hs775li were purchased from the Cell and Genome Engineering Core (CGEC) at the UCSF. Primary human melanocytic cell-lines (PHM) from infant foreskin of healthy donors were available in the Ortiz' lab cell repository.
Authentication	The cell lines were authenticated by the vendors.
Mycoplasma contamination	All cell lines tested were negative for mycoplasm contamination
Commonly misidentified lines (See ICLAC register)	No comonly misidentified cell lines were used.

Animals and other research organisms

Policy information about studies involving animals; ARRIVE guidelines recommended for reporting animal research, and Sex and Gender in Research

Laboratory animals	4- to 6-week-old homozygous nude Foxn1nu/Foxn1nu mice (Stock.no 007850). Mice were obtained from JAX®.
Wild animals	no wild animals were used
Reporting on sex	female mice were used.
Field-collected samples	no field-collected samples were used
Ethics oversight	Rodent experimental procedures were approved by the Office of Research institutional Animal Care and Use Program (IACUC, Chair: Jeremy Lieberman, MD) at the University of San Francisco (UCSF). All in vivo studies were conducted under the authorized protocol number AN174613-03.

Note that full information on the approval of the study protocol must also be provided in the manuscript.

Plants

Seed stocks	n/a
Novel plant genotypes	n/a
Authentication	n/a

Flow Cytometry

Plots

Confirm that:

- [ ] The axis labels state the marker and fluorochrome used (e.g. CD4-FITC).
- [x] The axis scales are clearly visible. Include numbers along axes only for bottom left plot of group (a 'group' is an analysis of identical markers).
- [x] All plots are contour plots with outliers or pseudocolor plots.
- [x] A numerical value for number of cells or percentage (with statistics) is provided.

Methodology

Sample preparation	1 x 10^5 D04 cells were seeded in six well-plates one day prior to transfection. One day after transfection live, dead, and apoptotic cells were differentiated using the Invitrogen™ Dead Cell Apoptosis Kits with Annexin V (cat.no.: V13241), following manufacturers protocol.
Instrument	BD® LSR II Flow Cytometer
Software	FACSDiva Software
Cell population abundance	Cells were harvested after one day, and stained for flow cytometry to determine the abundance of specific cell populations.
Gating strategy	Forward scatter (FSC) and side scatter (SSC) parameters were used to identify the overall cell population and exclude debris. A gate was drawn around the main cell population to include cells of interest based on size and granularity.

☐ Tick this box to confirm that a figure exemplifying the gating strategy is provided in the Supplementary Information.
